# Invasive snails, parasite spillback, and potential parasite spillover drive parasitic diseases of *Hippopotamus amphibius* in artificial lakes of Zimbabwe

**DOI:** 10.1186/s12915-021-01093-2

**Published:** 2021-08-20

**Authors:** Ruben Schols, Hans Carolus, Cyril Hammoud, Kudzai C. Muzarabani, Maxwell Barson, Tine Huyse

**Affiliations:** 1 Department of Biology, Royal Museum for Central Africa, Tervuren, Belgium; 2 Laboratory of Aquatic Biology, KU Leuven Kulak, Kortrijk, Belgium; 3 Laboratory of Molecular Cell Biology, KU Leuven-VIB Center for Microbiology, Leuven, Belgium; 4 Limnology Research Unit, Ghent University, Ghent, Belgium; 5 Department of Biological Sciences, University of Zimbabwe, Harare, Zimbabwe; 6 Department of Biological Sciences, University of Botswana, Gaborone, Botswana; 7 Lake Kariba Research Station, University of Zimbabwe, Kariba, Zimbabwe

**Keywords:** Trematodiasis_1_, Xenomonitoring_2_, One Health_3_, Barcoding_4_, Artificial lake_5_, Integrative taxonomy_6_, Taxonomic impediment_7_, Parasitology_8_, Conservation_9_, Biological invasions_10_

## Abstract

**Background:**

Humans impose a significant pressure on large herbivore populations, such as hippopotami, through hunting, poaching, and habitat destruction. Anthropogenic pressures can also occur indirectly, such as artificial lake creation and the subsequent introduction of invasive species that alter the ecosystem. These events can lead to drastic changes in parasite diversity and transmission, but generally receive little scientific attention.

**Results:**

In order to document and identify trematode parasites of the common hippopotamus (*Hippopotamus amphibius*) in artificial water systems of Zimbabwe, we applied an integrative taxonomic approach, combining molecular diagnostics and morphometrics on archived and new samples. In doing so, we provide DNA reference sequences of the hippopotamus liver fluke *Fasciola nyanzae*, enabling us to construct the first complete *Fasciola* phylogeny. We describe parasite spillback of *F. nyanzae* by the invasive freshwater snail *Pseudosuccinea columella*, as a consequence of a cascade of biological invasions in Lake Kariba, one of the biggest artificial lakes in the world. Additionally, we report an unknown stomach fluke of the hippopotamus transmitted by the non-endemic snail *Radix* aff. *plicatula*, an Asian snail species that has not been found in Africa before, and the stomach fluke *Carmyerius cruciformis* transmitted by the native snail *Bulinus truncatus*. Finally, *Biomphalaria pfeifferi* and two *Bulinus* species were found as new snail hosts for the poorly documented hippopotamus blood fluke *Schistosoma edwardiense*.

**Conclusions:**

Our findings indicate that artificial lakes are breeding grounds for endemic and non-endemic snails that transmit trematode parasites of the common hippopotamus. This has important implications, as existing research links trematode parasite infections combined with other stressors to declining wild herbivore populations. Therefore, we argue that monitoring the anthropogenic impact on parasite transmission should become an integral part of wildlife conservation efforts.

**Graphical abstract:**

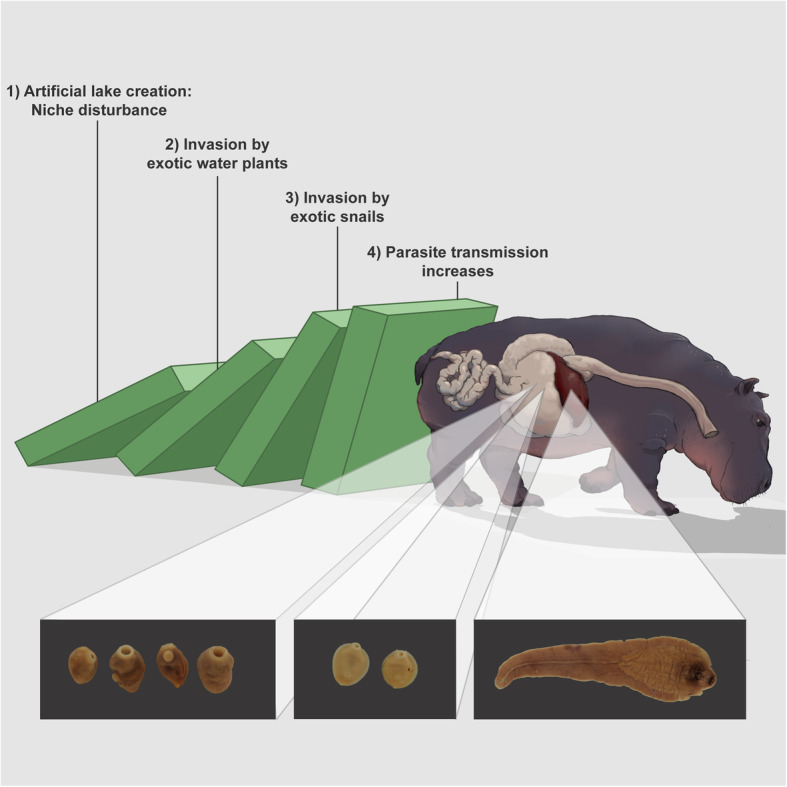

**Supplementary Information:**

The online version contains supplementary material available at 10.1186/s12915-021-01093-2.

## Background

Large artificial lakes can be a major driver of the socio-economic development of a region by improving water accessibility, stimulating agricultural irrigation and generating renewable energy [1, 2]. Globally, 3700 medium to large size artificial lakes (those with a capacity larger than 1 MW) have been built or are under construction [2, 3]. Nevertheless, dam construction also has tremendous repercussions on the surrounding environment as it can lead to the dislodgement and extinction of local aquatic communities, altered migration of aquatic biota, changes in aquatic biochemistry and physiology, disturbances of flood dynamics, introduction of exotic and invasive species, and the creation of breeding grounds for (non-)endemic vectors and intermediate hosts of (non-)endemic parasite species [4–11]. Additionally, the subsequent intensification of agriculture, animal husbandry and aquaculture near artificial lakes can lead to increased parasite transmission to humans, wildlife and domesticated animals [6, 12–15].

Snails play a key role as the intermediate host in the life cycle of trematode parasites and are a prime example of intermediate hosts that thrive in artificial lakes, as shown by multiple studies [5, 7, 10, 11, 16]. Consequently, the health burden on the definitive host of these parasites increases, as illustrated by the construction of the Diama dam in Northern Senegal, which caused drastic changes in the aquatic chemistry (e.g., reduced salinity and increased pH) that favored the colonization of planorbid snails and the subsequent establishment of one of the world’s biggest foci of human intestinal schistosomiasis [17]. Another study suggests that dams block migratory river prawns that predate on planorbid snails and thereby increase the risk of schistosomiasis for millions of people worldwide [16]. The use of agrochemicals such as fertilizers and pesticides on irrigated land can further stimulate snail—and thus trematode—prevalence by reducing snail predator populations and increasing algal growth, the main food source of freshwater snails [12, 15]. Besides favoring the colonization and proliferation of endemic snail species, artificial lakes can also facilitate the establishment of non-endemic snails that have the potential to drastically alter the dynamics of trematode transmission [5, 18, 19]. Invasive non-endemic snails can affect parasite transmission in three distinct ways: (1) by introducing non-endemic parasites, defined as “spillover”, (2) by successfully transmitting endemic parasites with an overall increased transmission as a result, defined as “spillback”, or (3) by transmitting endemic parasites but with a lower transmission potential, resulting in a “dilution” effect [20–22].

Recently, we found strong indications that in Lake Kariba, the world’s biggest artificial lake by volume, the proliferation of the invasive water hyacinth (*Eichhornia crassipes*) has facilitated the establishment of the invasive North American snail *Pseudosuccinea columella*, which in turn supports an extremely high prevalence (65%) of an unknown fasciolid trematode species [5]. The impressive success of *P. columella* snails in colonizing Lake Kariba, as well as the high trematode infection prevalence, suggests a significant health burden for the affected definitive host(s). The high parasite transmission by this invasive snail can be designated as either a spillback or a spillover phenomenon [22]. To discriminate between both scenarios, the identity of the parasite and final host are key. Carolus and colleagues [5] showed that the trematode transmitted by *P. columella* is phylogenetically closely related to *Fasciola gigantica* and *Fasciola hepatica.* However, the high genetic distances between this unknown species and other *Fasciola* species (based on mitochondrial cytochrome c oxidase subunit I (*COI*) sequences) suggested it to be (1) a new species, (2) a species not represented in current molecular databases, or (3) a hybrid species, as fasciolids are well-known for their hybridization potential [23–25]. Therefore, the aim of this study was to revisit Lake Kariba in order to collect more information on this parasite and its intermediate and definitive hosts. This subsequent sampling campaign involved the collection of snails and adult liver and stomach flukes from a culled hippopotamus, a prime suspect as definitive host [5]. An integrative taxonomic approach [26], combining nuclear and mitochondrial markers, was applied to reveal the identity of the unknown *Fasciola* sp. and other trematodes. In addition, we investigated whether similar phenomena of biological invasions coupled with parasite transmission are occurring in other artificial water systems in Zimbabwe.

## Results

### Trematode identification from the dissected hippopotamus

#### Morphological analysis

**Fig. 1.**
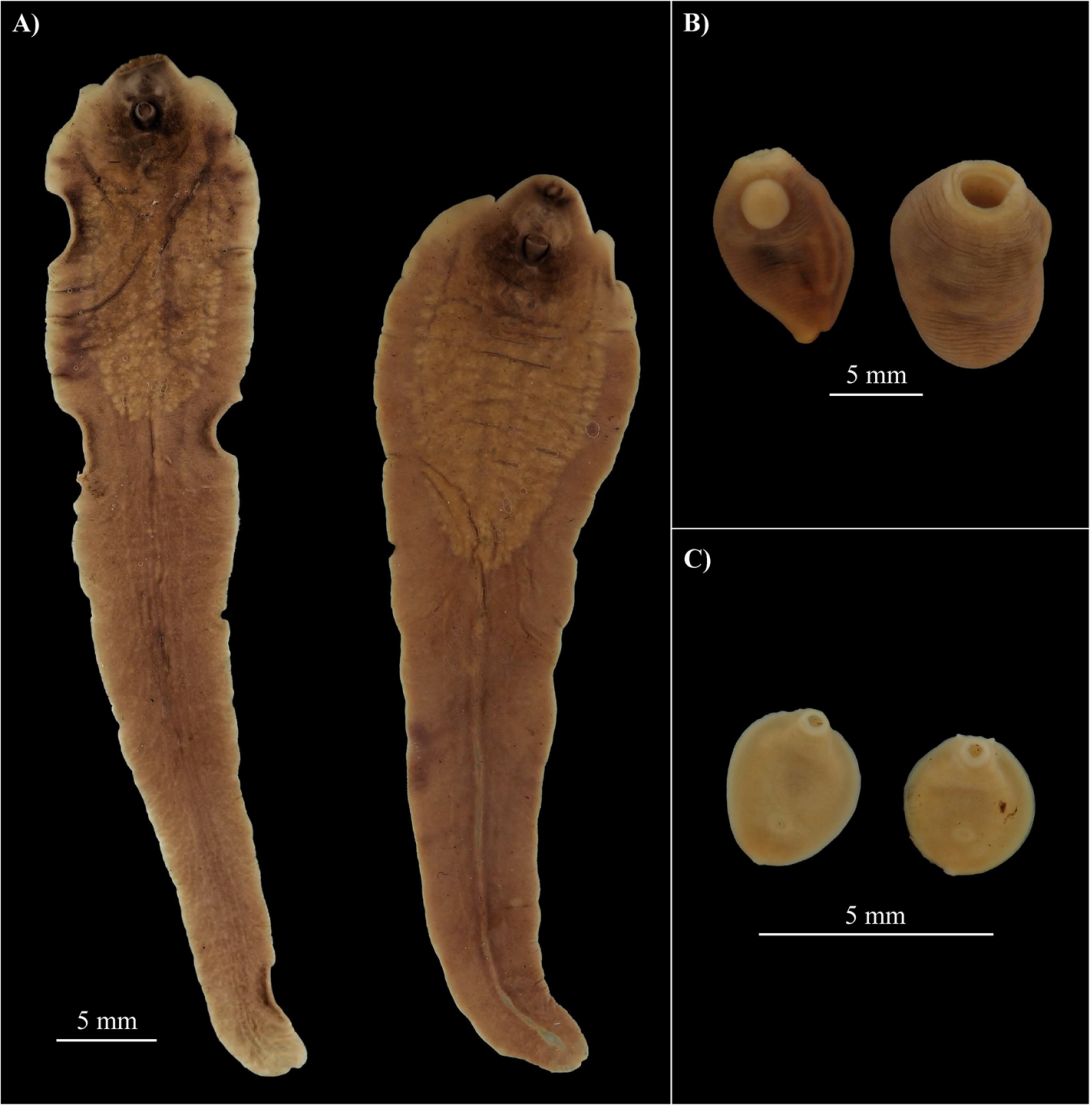
The collected flukes from the male hippopotamus at Lake Kariba. **A** Hippo liver flukes 1 and 2 (resp. left and right), **B** Hippo stomach fluke type 1, and **C** Hippo stomach fluke type 2. Scale bars represent 5 mm. Pictures were cropped and pasted to an artificial black background for contour visibility

Two morphologically distinct liver flukes (hereafter referred to as “Hippo liver fluke(s)”) were collected from the liver bile ducts of the culled hippopotamus. In addition, several hundred bright red stomach flukes (hereafter referred to as “Hippo stomach fluke type 1”) and two small yellowish stomach flukes (hereafter referred to as “Hippo stomach fluke type 2”) were collected from the stomach wall of the hippopotamus. A high-resolution photograph of each adult fluke morphotype is shown in Fig. 1. Scanning electron microscope (SEM) imaging was only possible for Hippo stomach fluke type 1 due to the low sample size of the other morphotypes (Additional file 1: Figure S1). Specimens of Hippo stomach fluke type 1 had a cylindrical shape that tapered towards the anterior end and showed a marked color change from bright red in fresh specimens to brown upon fixation. The Hippo stomach fluke type 2 had a discoid body shape and one specimen appeared to have two small caudal appendages (Fig. 1c, right specimen)*.* A comparative morphometric analysis (Table 1) of the fixed Hippo liver flukes described above and fixed museum specimens of *F. hepatica*, *F. gigantica*, and *F. nyanzae* (all shown in Additional file 1: Figure S2), combined with the descriptions in literature [27–29], indicates that the hitherto unknown *Fasciola* sp. present in Lake Kariba and Mwenje reservoir is morphologically most similar to *F. nyanzae.* The body width at 2 mm from the posterior end, the testis location, the testis to body length ratio, and the vitellaria morphology differentiate *F. nyanzae* from *F. hepatica* and *F. gigantica*. This, together with the fact that the definitive host is the common hippopotamus, supports the morphological identification of the Hippo liver fluke(s) as *F. nyanzae*. Additionally, measurements (Table 1) of the metacercariae (Additional file 1: Figure S3) that were released from two *P. columella* snails collected in Lake Kariba, fall within the range reported in literature for metacercariae of *F. hepatica*, *F. gigantica*, and *F. nyanzae* [27, 30, 31]. Notably, the observed shedding time between 9 and 11 pm (see the “Trematode infection prevalence in snails” section), coincides with the increased foraging activity of hippos at night [32].

**Table. 1:** Morphometric analysis of *Fasciola* species. Three reference measurements from literature are provided for *Fasciola nyanzae* collected from *Hippopotamus amphibius* [27–29]. All measurements are provided in mm, except measurements of metacercariae which are displayed in μm. Metacercarial measurements for Hippo liver fluke 1 and 2 were taken from two shedding *Pseudosuccinea columella* snails from site 3 in Kariba (Additional file 1: Figure S3). Metacercarial cyst measurements for *Fasciola gigantica* and *Fasciola hepatica* were taken from Alicata [30] and Vareille-Morel et al. [31], respectively. Variables that differentiate between the three *Fasciola* species are in bold italics. Measurements were taken with ImageJ software on 70% EtOH fixed samples with exception of the reports from literature. Measurement methodology is shown in Additional file 1: Figure S2. Abbreviations: body length to body width ratio (BL/BW), body length (BL), body width at widest point (BW), posterior width at 2 mm from the posterior end (BW2), cephalic cone length until shoulders (CL), ventral sucker anteroposterior diameter (VS), testis location in body length (Testis loc.), testis to body length ratio, vitellaria location in body length, outer diameter metacercaria (OD cyst), inner diameter metacercaria (without fibrous layer, ID cyst)

Species	Source	***Host***	BL/BW	BL	BW	***BW2***	CL	VS	***Testis loc.***	***Testis/BL***	***Vitellaria***	OD cysts (μm)	ID cysts (μm)
*F. nyanzae*	Leiper (1910)	*H. amphibius*	~ 7/1	69	~ 9	3	3	1.25	/	/	25	/	/
*F. nyanzae*	Jackson (1921)	*?*	3.6/1-7.8/1	35-59	4.5-13.5	/	/	/	Anterior third	/	/	/	/
*F. nyanzae*	Dinnik and Dinnik (1961)	*H. amphibius*	~ 7/1	49-91	~ 9	/	/	1.46-1.97	Anterior third	25%-40%	/	242-272	212-228
Hippo liver fluke 1	This study	*H. amphibius*	4.9/1	52.4	10.6	3.1	/	1.47	Anterior third	24%	34.1	261	221
Hippo liver fluke 2	This study	*H. amphibius*	3.5/1	46.9	13.3	3.2	2.2	1.72	Anterior third	29%	27.1	259	228
*F. nyanzae* RMCA	This study	*H. amphibius*	4.2/1	41.2	9.8	3	2.04	1.35	Anterior third	25%	25.4	/	/
*F. gigantica* RMCA	This study	Bovine	3.6/1	35.3	9.9	5.1	3.1	0.83	Anterior two third	41%	10.8	238-268	180-206
*F. hepatica* RMCA	This study	*Bos taurus*	3.1/1	26.8	8.6	4.6	1.67	1.47	Anterior two third	42%	10.16	205-256	/

#### Molecular analysis

**Fig. 2.**
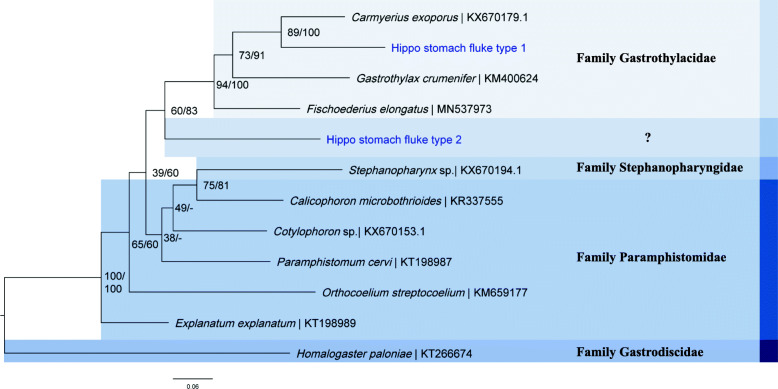
Maximum likelihood analysis of the available sequences of the Superfamily Paramphistomoidea using the GTR + G (= 0.80) + I (= 0.47) model on 758 bp of the *COI* marker. *Homalogaster paloniae* (“GenBank: KT266674”), from the family Gastrodiscidae, was used as an outgroup. Nodal support is indicated as bootstrap percentages (1000 bootstraps) and posterior probabilities, respectively before and after the “/” separator. Specimens from this study are indicated in blue. GenBank accession numbers are provided after the “|” separator

**Fig. 3.**
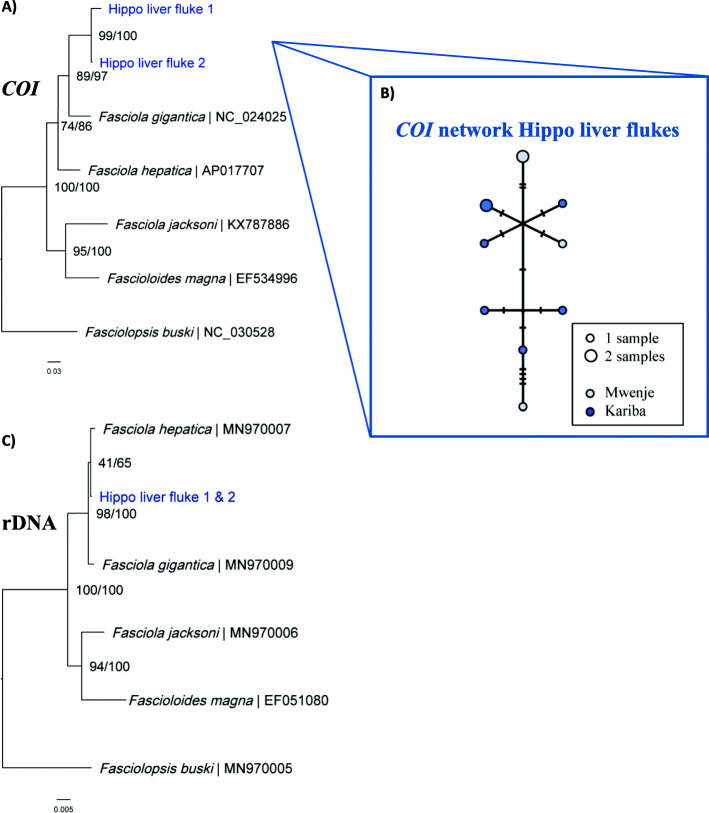
**A** Maximum likelihood analysis of the available sequences of the subfamily Fasciolinae using the GTR + G (= 0.25) model on 814 bp of the *COI* marker. *Fasciolopsis buski* (“GenBank: NC_030528”), from the subfamily Fasciolopsinae, was used as an outgroup. **B** The *Fasciola nyanzae COI* haplotype network of 11 sequences based on 412 positions, visualized using the TCS model in PopArt®. One hatch mark represents one mutation. The legend indicates the used color-code and haplotype abundance through circle size. **C** Maximum likelihood analysis of the available sequences of the subfamily Fasciolinae using the GTR + G (= 0.05) model on 2771 bp of the rDNA region. *Fasciolopsis buski* (“GenBank: MN970005”), from the subfamily Fasciolopsinae, was used as an outgroup. Nodal support is indicated as bootstrap percentages (1000 bootstraps) and posterior probabilities, respectively before and after the “/” separator. Specimens from this study are indicated in blue. GenBank accession numbers are provided after the “|” separator

The obtained *COI* sequences for Hippo stomach fluke type 1 and 2 (871 base pairs [bp] and 675 bp, “GenBank: MT909560 and MT909561” resp.) did not closely match any of the available sequences on the GenBank or BOLD databases. The closest BLAST hits were *Gastrothylax crumenifer* (89% identity score) and *Explanatum explanatum* (87% identity score), respectively. Our phylogenetic analyses show that Hippo stomach fluke type 1 has close affinity with the *Carmyerius* genus (Fig. 2). Additionally, the sequence proved 100% identical (*COI*, 376 bp) to an amphistome infection isolated from *Bulinus truncatus* in 2017 in Lake Kariba (“GenBank: MT013349 and MT013355” resp. for the snail and the amphistome infection; preprint, [33]). As no deeper taxonomic resolution could be obtained from the molecular data, we further refer to this species as “*Carmyerius* sp.” Based on *COI* sequencing, we can conclude that the Hippo stomach fluke type 2 belongs to the superfamily Paramphistomoidea, but it could not be identified to a lower taxonomic level in our phylogenetic analysis (Fig. 2). The *COI* sequence was, however, 100% identical (420 bp) to an amphistome infection in *Radix* sp. (“GenBank: MT013350”; preprint, [33]), here identified as *Radix* aff*. plicatula* (see the “Snail identification” section), from Lake Kariba [[5]; preprint, [33]]. All pairwise distances with *COI* reference sequences on GenBank exceed 12% and thereby surpass the 10% divergence of congeneric trematodes [34]. Therefore, we further refer to this species as “unidentified amphistome species”. DNA sequencing of Hippo liver fluke 1 and 2 resulted in *COI* fragments of 815 and 862 bp (“GenBank: MT909542 and MT909543” resp.) and rDNA fragments of 3083 and 2987 bp (“GenBank: MT909821 and MT909820”), respectively. The rDNA region, which was identical between the two flukes, covered almost the entire *18S* rDNA (1829 bp, see the “Methods” section and Additional file 1: Figure S4), internal transcribed spacer 1 (*ITS1*, 454 bp), *5.8S* rDNA (160 bp), internal transcribed spacer 2 (*ITS2*, 360 bp), and partial *28S* rDNA (281 and 185 bp resp. for Hippo liver fluke 1 and 2) regions. The *COI* sequences differed 2.2% over 814 nucleotides. The *COI* and rDNA sequences showed the highest similarity (twice 99.3% for COI [“GenBank: MK330628-MK330630”] and 99.9% for rDNA [“GenBank: MK330623-MK330625”]) to the *Fasciola* sp. infections in *P. columella* and *Radix* sp. (here identified as *R.* aff*. plicatula*, see the “Snail identification” section) from Lake Kariba, reported by Carolus et al. [5]. The COI pairwise distances of Hippo liver fluke 1 and 2 with *F. gigantica* (10.6% and 14.4%, resp.) and *F. hepatica* (16.4% and 18%, resp.) exceed the 5% threshold for species differentiation based on the *COI* barcoding marker [34, 35]. The rDNA pairwise distances (rDNA sequences were identical between Hippo liver fluke 1 and 2) to *F. gigantica* and *F. hepatica* were less pronounced compared to *COI* distances (0.4% and 0.2%, resp.) (Additional file 1: Table S1 and S2). Phylogenetic analysis based on *COI* sequences place the Hippo liver fluke as a sister taxon to *F. gigantica* within the *Fasciola* genus (Fig. 3a and Additional file 1: Table S1). The placement in the *Fasciola* genus is confirmed by the rDNA-based phylogeny (Fig. 3c and Additional file 1: Table S2). No DNA sequences could be obtained from the museum samples, probably due to DNA degradation or PCR inhibition as a result of long-term preservation, first in formaldehyde and later in 70% ethanol. This prevented the inclusion of the museum samples (shown in Additional file 1: Figure S2) in our phylogenetic analysis. None of the tested datasets showed conclusive evidence of substitution saturation (see supplementary file: “Raw_data” for the Steel’s and Xia’s method outputs per dataset).

### Snail identification

**Fig. 4.**
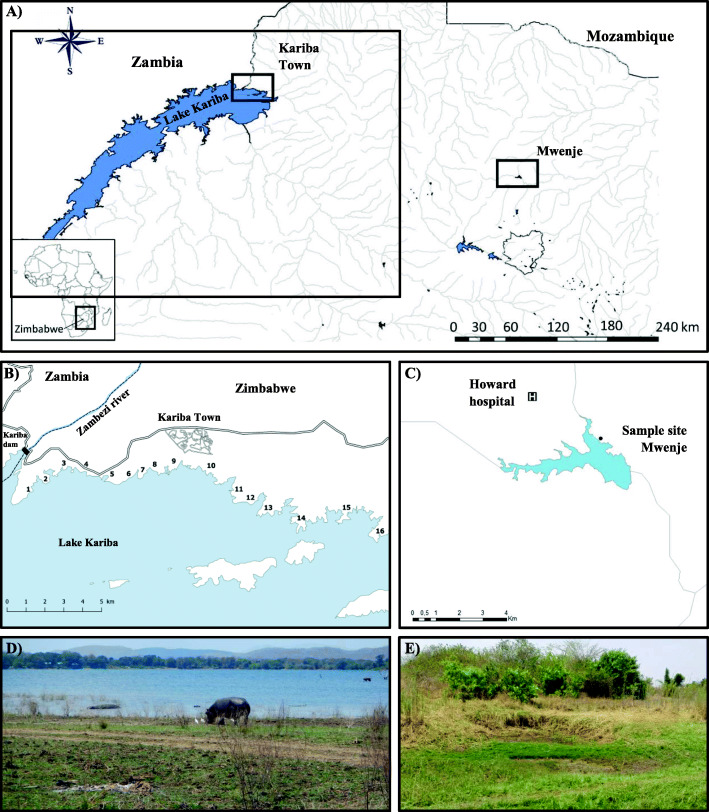
**A** Map indicating the sampling locations in the Zambezi basin including “Kariba Town” at Lake Kariba and “Mwenje” at Mwenje reservoir in Zimbabwe. **B** Sampling sites at Lake Kariba; figure adapted from Carolus et al. [5]. **C** Sampling site at Mwenje, a temporary puddle adjacent to the main reservoir located at S 17°14′ 47.9″ E 31°01′ 07.7″; figure adapted from Schols et al. [41]. **D** Sampling site 13 at Lake Kariba. **E** Sampling site at Mwenje reservoir

**Fig. 5.**
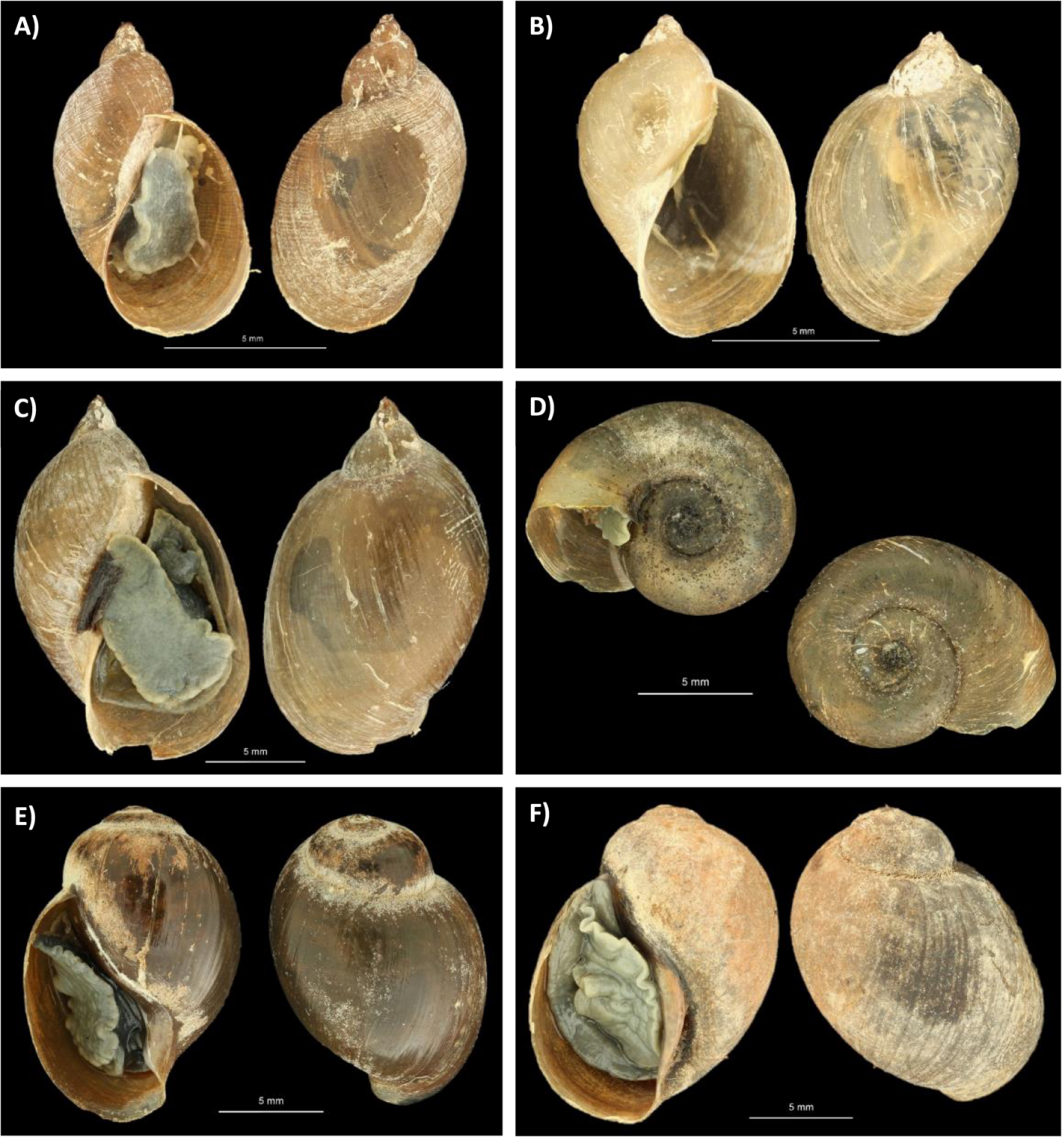
Pictures of snail morphotypes collected in Mwenje and Kariba. **A** *Pseudosuccinea columella*, **B** *Radix* sp. molecularly identified as *Radix* aff*. plicatula*, **C** *Radix natalensis*, **D** *Biomphalaria pfeifferi*, **E** *Bulinus globosus*, and **F** *Bulinus* sp. Scale bars represent 5 mm. Pictures were cropped and pasted to an artificial black background for contour visibility

Six different snail species were sampled in this study: *Pseudosuccinea columella* and *Radix* aff*. plicatula* at Lake Kariba and *Radix natalensis*, *Biomphalaria pfeifferi*, *Bulinus globosus*, and *Bulinus* sp. at Mwenje reservoir (Fig. 4). Out of 16 sampled sites in Lake Kariba, only site 3 and site 16 harbored snails (see Table 2 for the number of snails per site and Fig. 4 for information on sampling locations). High-resolution photographs of one fixed specimen per species are depicted in Fig. 5.

**Table. 2:** Snail abundance and trematode infections per site. All snail species are listed per site along with the number of specimens collected (Sampled), the number of snails that shed cercariae in the shedding experiment (Shedding), the number of snails for which DNA was extracted for diagnostic multiplex PCRs (Tested), the number of samples that tested positive for a trematode infection (Trematoda inf.), and how many of those trematode infections had a *Fasciola nyanzae* (*F. nyanzae* inf.), *Schistosoma edwardiense* (*S. edwardiense* inf.), or *Schistosoma haematobium* infection (*S. haematobium* inf.). For Kariba sites, the number between brackets refers to exact sample location adopted from Carolus et al. [5]. High-resolution pictures of each snail morphotype are shown in Fig. 5

Site	Species	Sampled	Shedding	Tested	Trematoda inf.	*F. nyanzae* inf.	*S. edwardiense* inf.	*S. haematobium* inf.
Kariba (3)	*Pseudosuccinea columella*	60	6	24	21	21	0	0
Kariba (3)	*Radix* aff*. plicatula*	12	0	12	3	1	0	0
Kariba (16)	*Pseudosuccinea columella*	43	0	24	24	24	0	0
Mwenje	*Radix natalensis*	17	0	17	13	11	0	0
Mwenje	*Biomphalaria pfeifferi*	6	0	6	6	0	3^a^	0
Mwenje	*Bulinus* sp.	1	0	1	1	0	1	0
Mwenje	*Bulinus globosus*	9	0	9	9	0	6	1

^a^One more *B. pfeifferi* showed infection signals indicating a *Schistosoma* sp. infection but no sequences could be obtained, inhibiting species identification

*COI* sequences of *P. columella* were 100% identical (463 and 433 bp, “GenBank: MT888842”) to the invasive *P. columella* haplotype reported across Africa, Australia, and Northern America (i.e., Egypt [“BOLD: GBMIN110283-17”], Australia [“BOLD: GBMLG0711-06”], and USA [“BOLD: GBMPL484-13”]). We confirm the presence of this invasive haplotype in Lake Kariba, as published before [5] (see “GenBank: MK333465”). Carolus and colleagues [5] reported an unidentified *Radix* sp., presumably originating from Asia, but the authors were not able to identify the species, as closely related reference sequences were missing at that time. The *COI* sequences from the *Radix* species sampled in this study (“GenBank: MT888847”) show only one mutation in the 350 bp overlapping *COI* sequence compared to the *Radix* sp. sequenced by Carolus et al. [5] (see “GenBank: MK333466”). In our phylogenetic analyses, the *Radix* sp. shows well supported clustering with a recently generated reference sequence of *Radix plicatula* from China [37] (Additional file 1: Figure S5 and Table S3). However, many radicine snail species are absent from online genetic databases, and shell morphology differs from the description in Vinarski et al. [38]. Therefore, and in combination with the pairwise distance of 3.6% between the *Radix* sp. from Kariba and the reference sequence of *R. plicatula* on a 463 bp *COI* alignment, we identify our current specimen and the previously reported *Radix* sp. from Lake Kariba as the non-endemic *R. affinis plicatula* (Additional file 1: Table S3). The qualifier *affinis* is utilized in accordance with the open nomenclature guidelines of Sigovini and colleagues [39], as our specimens show clear molecular affinity to *R. plicatula*, but are not identical to it.

The identity of *R. natalensis*, *B. pfeifferi*, and *B. globosus* collected in Mwenje (446 bp, 446 bp, and 463 bp resp.; “GenBank: MT888844-MT888846”) was confirmed based on phylogenetic clustering and pairwise distances below 5%, compared to *COI* reference sequences in the BOLD and NCBI databases (Additional file 1: Figure S5, S6 and Table S3, S4 and S5). A second *Bulinus* species from Mwenje (446 bp, “GenBank: MT888843”) could not be identified to species level based on *COI* barcoding but appears to be part of the *Bulinus truncatus/tropicus* species complex as indicated by the firm clustering within this group in our phylogenetic analysis (Additional file 1: Figure S6a and Table S5). Further taxonomic identification was not possible as the species group contains 14 species that are morphologically nearly indistinguishable, many of which still lack reliable reference genetic material [36, 40].

### Trematode infection prevalence in snails

Cercarial shedding (i.e., release of trematode larvae) was exclusively observed between 9 pm and 11 pm in six out of 103 examined *P. columella* snails (5.8%) collected in Kariba. No other snail species was found to release cercariae during shedding experiments. When screening for infections through diagnostic PCRs, more infected snails were detected. Table 2 shows the number of snails tested, the number of trematode infections detected and the number of samples where a *Schistosoma* sp. or *Fasciola* sp. infection was detected in the multiplex PCR assay. In summary, 77 out of 93 samples (82.8%) had (pre)patent trematode infections. Of all detected infections, a total of 57 out of the 61 (93.4%) infected lymnaeid snails (*P. columella* and *R.* aff*. plicatula* from Kariba and *R. natalensis* from Mwenje) were infected with a *Fasciola* sp., while 12 out of the 16 (75%) infected planorbid snails (*B. pfeifferi*, *B. globosus*, and *Bulinus* sp. from Mwenje) were infected with a *Schistosoma* species.

### Trematode identification from snail infections

Tissue of one *P. columella* specimen and one *R.* aff*. plicatula* specimen from Lake Kariba with a positive *Fasciola* sp. PCR signal was used for trematode *COI* barcoding (425 bp and 482 bp resp., “GenBank: MT909545 and MT909549” resp.). Both *COI* sequences showed a pairwise distance of less than 0.5% compared to each other and less than 0.8% compared to the two adult *F. nyanzae* specimens from the culled hippopotamus (based on 408 bp). Eleven specimens of *Radix natalensis* from Mwenje with a positive *Fasciola* sp. PCR signal were used for *COI* and *ITS1-5.8S-ITS2* sequencing. Five *COI* sequences (~ 400 bp) were ~ 99% identical to a *Diplostomidae* sp. collected from the adjacent reservoir by Schols et al. [41] (“GenBank: MT994279”), two did not provide sequencing results of sufficient quality and the remaining four amplicons (424 bp–428 bp, “GenBank: MT909546-MT909548 and MT909550”) showed a pairwise distance below 2% to the two adult *F. nyanzae* specimens from Kariba (results not shown, 408 bp). The *ITS1*-*5.8S*-*ITS2* amplification and sequencing was successful for 10 out of the 11 *Fasciola* sp. positive samples (464 bp, “GenBank: MT893586-MT893595”). All 10 sequences were 100% identical to each other, to the two adult *F. nyanzae* and to the S151 haplotype from Carolus et al. [5] (“GenBank: MK330624”).

Using the two-step diagnostic PCR assay of Schols et al. [42] (see the “Methods” section, “detection of infected snails”), we identified *S. haematobium* in a *B. globosus* snail*.* This was confirmed by sequencing part of the *COI* marker (335 bp, “GenBank: MT886703”) and rDNA region (985 bp, “GenBank: MT884914”) in this specimen*.* The remaining *Schistosoma* sp. infections in *B. pfeifferi* (4), *B. globosus* (6) and *Bulinus* sp. (1) gave a *Schistosoma-*specific amplicon in the first multiplex PCR but did not yield a species-specific amplicon in the second multiplex PCR, indicating it was not *Schistosoma haematobium*, *Schistosoma mansoni*, *Schistosoma mattheei*, *Schistosoma bovis*, *Schistosoma curassoni*, or *Schistosoma guineensis* [42]. We, therefore, sequenced the *COI* marker and the *Schistosoma*-specific *5.8S*-*ITS2* amplicon generated in the first multiplex PCR. The *COI* marker (335 bp, “GenBank: MT886702”) was successfully amplified for only one *B. pfeifferi* infection and showed a pairwise distance of 0.2% to the preliminary identified *S. edwardiense* isolated from *Biomphalaria sudanica* in Uganda (“GenBank: AY197347”). The *5.8S*-*ITS2* (~ 340 bp, “GenBank: MT884915-MT884924”) marker was successfully sequenced for ten out of the eleven remaining samples and all sequences were 100% identical to each other and to the *S. edwardiense* reference sequence (“GenBank: AY197344”). Based on these results, we identify all ten remaining *Schistosoma* sp. infections in *B. pfeifferi* (3), *B. globosus* (6), and *Bulinus* sp. (1) as *S. edwardiense.*

### Haplotype network of *Fasciola nyanzae*

The genetic diversity of *F. nyanzae* in Lake Kariba and Mwenje reservoir was assessed using all available *COI* sequences of *F. nyanzae.* To build the haplotype network, we included both adult liver flukes collected from the hippopotamus, *F. nyanzae* sequences isolated from four infected *P. columella* from Lake Kariba (of which one from this study and three from Carolus et al. [5]: “GenBank: MK330623-MK330625”), one sequence from an infected *R.* aff*. plicatula* from Lake Kariba and *F. nyanzae* sequences from four infected *R. natalensis* from Mwenje reservoir. The resulting alignment (412 bp) consisted of 11 sequences, of which nine were unique, and the associated haplotype diversity (Hd) was 0.96. The highest pairwise distance between two haplotypes was 1.7%. The haplotype network shows no spatial genetic structure (Fig. 3b).

All GenBank identifiers generated by this study, and those of Carolus et al. [5] and Muzarabani et al. (preprint, [33]) involved in the analyses, are listed together with the sample type, marker, taxonomic identification and region of origin in Additional file 1: Table S6.

## Discussion

In this study, we report four trematode species that infect the common hippopotamus and show that they are transmitted by six snail species that thrive in Lake Kariba and Mwenje reservoir. We are able to complete the life cycle for three of these species by identifying them in both the final and the intermediate host. Below, we discuss the biological phenomena behind parasite transmission in artificial lakes and the possible implications for threatened hippopotamus populations.

### 1. Completing the *Fasciola* phylogeny

The adult liver flukes sampled in this study match the morphological descriptions of *Fasciola nyanzae* made by Leiper in 1910 [27]. Since then, only two scientific reports have elaborated on the morphology and life cycle of *F. nyanzae* [27, 28]. Along with morphological measurements of the cercariae that were genetically identical to the adult liver flukes, these morphological descriptions enable us to identify *F. nyanzae* and provide the first genetic record of this species. As a result, we can provide the last missing link in the molecular phylogeny of the *Fasciola* genus and show that *F. nyanzae* is most closely related to *Fasciola gigantica* based on partial *COI* mtDNA (Fig. 3a and Additional file 1: Table S1). This close relationship is also supported by the observation that both species can infect the intermediate snail host *R. natalensis* and the definitive mammalian host *H. amphibius*, while no records exist of *Fasciola hepatica* in hippopotami [27, 43]*.* This phylogenetic relationship is, however, less clear based on the more conserved nuclear markers, where the distances between *F. nyanzae* and both *F. hepatica* and *F. gigantica* are of the same order and below 1% (Fig. 3c and Additional file 1: Table S2). This apparent mito-nuclear discordance can either be explained by drastically different evolutionary rates between nuclear and mitochondrial DNA [44] or by introgressive hybridization [24, 45]. A higher genomic coverage is needed to conclude on this, as concerted evolution in the rDNA region can blur biological phenomena, such as a bias towards one of the two parental species after hybridization [46]. Our phylogenetic analyses also enable us to contribute to the taxonomic discussion on whether or not *Fasciola jacksoni* should be reclassified as *Fascioloides jacksoni*, as suggested by Lotfy et al. [47]. Mas-Coma et al. [43] opposed this reclassification, because the reference sequences used by Lotfy et al. [47] originated from regions where *F. gigantica x F. hepatica* hybrids are known to occur, *F. nyanzae* and *Tenuifasciola tragelaphi* were missing from the phylogenetic analysis, and the mitochondrial marker (*nad1*) used in the study of Lotfy et al. [47] is too variable, leading to substitution saturation. The inclusion of well-selected reference sequences from regions where no hybridization has been reported, the addition of *F. nyanzae*, and the use of a less variable mtDNA marker [48] in this study, results in a dataset without substitution saturation and resolves most of the aforementioned issues. Our maximum likelihood and Bayesian phylogenies of mitochondrial and nuclear markers strongly support the position of *F. jacksoni* within the genus *Fascioloides* (Fig. 3a and c and Additional file 1: Table S1 and S2, respectively).

### 2. Parasite spillback of the hippo liver fluke *Fasciola nyanzae* in Lake Kariba

*Fasciola nyanzae* infections were detected in three lymnaeid snail species: the endemic *Radix natalensis*, the Asian *Radix* aff*. plicatula*, and the North American *Pseudosuccinea columella*. The latter two species are invasive non-endemic snails that could affect parasite transmission in three distinct ways: parasite spillover, parasite spillback or a dilution effect (see the “Background” section for explanations). Co-invading parasites, like other invading fauna and flora, typically go through a genetic bottleneck, resulting in low genetic diversity in the invasive population [49]. In contrast, our *F. nyanzae* samples show a high genetic diversity with 9 out of the 11 sequenced *COI* haplotypes being unique. Moreover, their definitive host (*H. amphibius*) and at least one of their intermediate hosts (*R. natalensis*) are endemic to Zimbabwe [50, 51], supporting the endemicity of *F. nyanzae* to this region. In addition, another requirement to comply with a “parasite spillback” hypothesis was met, as a higher *F. nyanzae* infection prevalence was detected in the invasive snail *P. columella* compared to native snails. We can therefore confirm that the cascade of biological invasions, in which the invasion of *P. columella* was facilitated by the introduction and subsequent colonization of water hyacinth from South America as described in Carolus et al. [5], has led to “parasite spillback” of the endemic parasite *F. nyanzae* in Lake Kariba. Water hyacinth [52] and lymnaeid snails such as *P. columella* [53] generally thrive in nutrient rich, still, or slow-moving water, but not in fast moving riverine systems, like the Zambezi river, which occupied the Kariba gorge before the construction of Kariba dam. Therefore, we hypothesize that the parasite spillback phenomenon we witness here, is a result of this man-made impoundment.

### 3. Two hippopotamus stomach flukes, one case of parasite spillover?

Although amphistomiasis, or stomach fluke disease, is one of the most prevalent and pathogenic animal trematodiases, there is a significant knowledge gap concerning the life cycle, host compatibility, prevalence, and geographic range of many amphistome species, especially in Africa [54–56]. Amphistomes are most commonly studied in livestock, especially ruminants, but many species infect wild animals. Here, we provide sequences, the intermediate host identity, and high–resolution photographs of two amphistome species of the common hippopotamus (i.e., *Carmyerius cruciformis* and an unidentified amphistome species). We link these stomach fluke species to infections in the endemic snail *B. truncatus* reported by Muzarabani et al. (preprint, [33]) and the non-endemic snail *R.* aff*. plicatula*, respectively. Based on its morphology (Fig. 1b and Additional file 1: Figure S1), its final and intermediate hosts and close phylogenetic affinity to *Carmyerius exoporus* (Fig. 2), we tentatively identify the amphistome Hippo stomach fluke type 1, genotyped as “*Carmyerius* sp.” in this study, as *Carmyerius cruciformis*. First of all, the reported size, shape, and the distinct color change upon fixation in our specimens match prior descriptions [29, 57]. Secondly, it is the only *Carmyerius* species described from the hippopotamus so far [58]. Thirdly, members of the *Bulinus* genus are known intermediate hosts of this parasite [59], which corresponds with the prior infections detected in *B. truncatus* (“GenBank: MT013350, MT013355”; preprint, [33]), that match our genotype. Fourthly, the oral opening and tegumental papillae (Additional file 1: Figure S1) correspond to prior reports of *C. cruciformis* [57, 60]*.* And finally, no reference sequence exists of *C. cruciformis*, probably explaining the failed attempt to identify this species through molecular barcoding*.* To our knowledge, this is the first molecular record and southernmost report (other reports are from Kenya, Uganda, Benin, and Chad [58]) of *C. cruciformis*.

The amphistome Hippo stomach fluke type 2 (Fig. 1c) could not be identified based on the *COI* phylogeny that includes all families of the superfamily Paramphistomoidea present in online molecular databases (Fig. 2). The absence of a morphologically similar hippopotamus trematode described in literature and the lack of reference sequences for many amphistome families (e.g., family Brumptinae) make the identification of these specimens currently impossible. To further elucidate its phylogenetic position and identify it to species level, future research should study the morphological characteristics such as the tegumental surface and the genital pore through scanning electron microscopy [60] and transversal sections [57], respectively. Furthermore, an integrative taxonomic approach could be applied to generate reference sequences for the missing amphistome families [26, 41]. For the Hippo stomach fluke type 2, sampled from the Asian snail *R.* aff*. plicatula*, no prior genetic and morphological records exist, which raises the question whether this parasite is endemic to Africa. The answer will determine whether this is a case of a potential “parasite spillback” (like for *F. nyanzae*), or a “parasite spillover,” as a result of co-introduction of a non-endemic parasite with a non-endemic snail host.

### 4. New intermediate hosts for the hippopotamus blood fluke *Schistosoma edwardiense*?

Three endemic planorbid snail species, namely *B. pfeifferi*, *B. globosus*, and an unidentified *Bulinus* sp. from the *Bulinus truncatus/tropicus* complex, were found to be infected with the hippopotamus blood fluke *S. edwardiense.* Although schistosome blood flukes are among the most studied trematode species due to their public health importance, wildlife schistosomes are severely understudied. *S. edwardiense* has so far only been reported in Uganda [61] and South Africa [62]. The species is known to be transmitted through *Biomphalaria* snails [61], and so far, *Bulinus* snails have not been reported as an intermediate host*.* We cannot conclude whether the molecular detection of *S. edwardiense* in the two *Bulinus* species reflects true, compatible host-parasite combinations or whether they are a result of aborted infections, since we did not observe cercarial shedding in these samples (neither in the infected *Biomphalaria* snails). However, we found a positive PCR signal in seven bulinid specimens, and the closest known relative of *S. edwardiense*, *Schistosoma hippopotami*, is also transmitted through bulinid snails [61]. These facts strengthen our hypothesis that apart from *B. pfeifferi*, bulinid snails are compatible hosts for *S. edwardiense* too. Our results strengthen the statement of Morgan et al. [61] that *S. edwardiense* and *S. hippopotami* belong to a different group, ancestral to the *S. mansoni* and *S. haematobium* species groups. The ability to use members of both the *Biomphalaria* and *Bulinus* genera appears as a “primitive” trait, which is lost in the derived species groups that specialized in only one of the two snail genera, respectively.

### 5. The potential health burden of hippopotamus trematodiases

Trematode parasites can have detrimental effects on the health, lifespan, and reproduction of wild and domestic animals. For instance, liver flukes cause hepatic fibrosis, cirrhosis, internal hemorrhage, and calcification of the bile duct [63]. As a result, fascioliasis in livestock is associated with an increased mortality and abortion rate, reduced growth and a lower productivity [64, 65], which accounts for global economic losses exceeding 3.2 billion US dollars [64]. *Fasciola jacksoni* and *Protofasciola rubusta* can form a major health burden and source of (calf) mortality in Asian [66] and African [67] elephants, respectively. Additionally, *F. jacksoni* has been suggested to be a major contributor to the drastic decline of endangered elephant populations in Asia [66]. Another fluke, *Fascioloides magna*, is thought to be the single major cause of mortality in a declining population of moose in the USA [68]. Schistosomes or blood flukes are also thought to be an important yet overlooked cause of animal mortality and productivity losses in the livestock industry, with a major underestimated economic impact [69]. Certain schistosome species have also been suggested to be of concern to conservation of endangered animals such as rhinos and elephants in Asia [70] and chimpanzees in Africa [71]. In addition to liver and blood flukes, immature stomach flukes, or amphistomes, inflict damage to the intestine, leading to anorexia and diarrhea, while severe infections can be lethal [72, 73]. Amphistome infections are regarded as one of the most prevalent parasitic disease in livestock [55]. Due to their high morbidity and mortality in young animals, some authors hypothesize that the economic losses due to amphistomiases are greater than many other parasitic diseases in livestock [74].

Few records exist on the pathology and impact of trematode infections in hippopotami, but a similar impact as described above can be expected. The most elaborate study on hippopotamus trematodiases dates back to 1967 and reports one hundred culled hippopotami of the Kruger National Park in South Africa [75]. It describes unknown fasciolids that colonized the bile duct in large numbers, causing lesions and fibrosis of the duct wall and damage extending into the liver parenchyma. The same study reports a high load of schistosomes in the heart and most major blood vessels, especially in the venae cavae, pulmonary and hepatic veins, associated with inflammation, macroscopic lesions, and endocarditis [75]. Since then, no further pathological reports on trematode infections in the common hippopotamus have been published to the best of our knowledge.

Here, we provide a parasitological survey of a single culled hippopotamus from Lake Kariba, in which only parts of the liver and stomach lining were studied. This revealed three different trematode species (i.e., *F. nyanzae*, *C. cruciformis* and an unidentified amphistome) with a very high infection intensity of the stomach fluke *C. cruciformis*. In addition, the high trematode infection prevalence in snail intermediate hosts, reported here and in Carolus et al. [5], indicates that the exposure risk of hippopotami to these parasites is high.

### 6. Do artificial lakes and biological invasions pose a burden on *Hippopotamus amphibius*?

Considering our findings and the aforementioned burden of trematodes on wildlife populations, we argue that artificial lakes can act as a breeding ground for (non-)endemic intermediate snail hosts that transmit hippopotamus infecting parasites, and thereby indirectly affect hippopotamus (and possibly other wildlife) populations. Nevertheless, the actual impact of dam construction on hippopotami cannot be directly verified. No data is available on hippopotamus populations and their trematode fauna prior to dam construction or the arrival of invasive snail species. However, key historical reports do show a link between the Kariba dam construction and altered snail and trematode dynamics in the area. A health risk assessment prior to the Kariba dam construction concluded that the chance for a schistosomiasis outbreak was minimal given the rocky substrate of Kariba gorge, which is unsuitable for freshwater snails [76]. At that time, only urinary schistosomiasis was present in the surrounding communities at low prevalence, while *B. pfeifferi* and thus intestinal schistosomiasis was absent. However, soon after dam construction, urinary schistosomiasis increased and for the first time, intestinal schistosomiasis was reported [77]. Furthermore, *Bulinus*, *Biomphalaria*, and lymnaeid species, all potentially suitable intermediate snail hosts for the hippopotamus parasites we describe here, colonized the lake [77]. Later studies confirmed the presence of planorbid snails and increased levels of schistosomiasis in nearby towns [78–81]. Ultimately, the invasive snails *P. columella* and *Radix* sp*.* (here identified as *R. aff. plicatula*), which we find to be compatible intermediate hosts for hippopotamus parasites, were first reported in the lake in 2017 [5]. In conclusion, these historical records and our findings strongly suggest that the creation of Lake Kariba has drastically altered parasite transmission dynamics by favoring the colonization and proliferation of (non-)endemic snail species that transmit trematode parasites, including those infecting hippopotami.

Large herbivores such as hippos play pivotal roles in their ecosystems and are therefore crucial targets for conservation efforts, which rely on a profound understanding of the factors that put the target population at risk [82–85]. Among the major anthropogenic disturbances, such as habitat loss, hunting, and poaching [82, 86], the impact of parasitic diseases remains hard to assess due to knowledge gaps in the diversity and epidemiology of most wildlife parasites [87]. Therefore, the impact of trematode parasites on wildlife populations might be severely underestimated [88]. The common hippopotamus is listed as vulnerable to extinction [50], yet remains populous in southern and eastern Africa with an estimated 5000 specimens in Zimbabwe [50, 85]. However, models show that these populations are two to five times more likely to cross the risk threshold of drastic decline in the next 60 years, if mild human-induced perturbations continue, alongside the present natural disturbance [85]. The combination of disease with other environmental or anthropogenic stressors can impose significant conservation threats [88–90]. This is illustrated by Obanda et al. [91], which suggest that infections with the liver fluke *P. robusta* have a synergetic effect with drought-related starvation in mass-mortality of African elephants in Kenya. Parasitic diseases can thus have major consequences for threatened populations, making it a critical, although understudied issue for the conservation of species [92].

## Conclusions

The data presented here suggests that the construction of artificial lakes along with the introduction and establishment of endemic and non-endemic species might have increased or at least altered the burden of snail-borne diseases on hippopotami. Still, to quantify what the exact impact might be, we lack historic and current information on the prevalence and infection intensities of trematode species in the hippopotamus populations in this region. Such information is hard to obtain and requires collaborations between researchers in the fields of ecology, veterinary science, and conservation biology as suggested by previous studies [92, 93]. Nevertheless, enriching the genetic database of wildlife trematodes to design non-invasive sampling methods can generate valuable resources in conservation studies [92]. For example, by providing the genetic reference of parasites like *F. nyanzae* and *C. cruciformis*, its prevalence can be monitored by designing species-specific diagnostic PCRs to screen stool samples from hippos and other wildlife species.

## Methods

### Adult fluke collection and morphometric characterization

A subadult male *Hippopotamus amphibius* was culled by rangers as part of the wildlife governance quota set for population control, problem animal management, community benefits, or other aspects of sustainable utilization by Zimbabwe Parks and Wildlife Management Authority. The hippopotamus was culled near Kariba Town (Fig. 4), and the liver, bile ducts, and stomach were dissected and inspected for adult trematode parasites. The entire liver and a 15 by 15 cm part of the stomach were screened for liver and stomach flukes, respectively. Fresh adult flukes were stored in 70% ethanol for later DNA analysis and morphometrics. The fixed flukes collected in this study, along with museum (Royal Museum for Central Africa, RMCA) specimens of *Fasciola nyanzae*, *F. gigantica*, and *F. hepatica* (“RMCA tissue vouchers: 31048, 29430, and 22595, resp.”), that were collected in the Democratic Republic of Congo in 1956, 1952, and 1938, respectively, were photographed using a Canon EOS 600D camera equipped with a Macro Photo Lens according to Brecko et al. [94]. Pictures were taken at different focal depths while the specimens remained submerged in 70% ethanol and stacked using the Zerene Stacker™ software. Stacked pictures of the adult flukes were used for measurements in the ImageJ software (version 1.8.0). Morphometric measurements were taken of body width at the widest point (BW), body length (BL), cephalic cone length until shoulders (CL), width at 2 mm from the posterior end (BW2), and the anteroposterior diameter of the ventral sucker (VS) (see Additional file 1: Figure S2 for a detailed visualization of how the measurements were done). Specimens for scanning electron microscopy were transferred in acetone for 24 h, then airdried, mounted on aluminum stubs, coated with gold, and studied using a JEOL JSM-6480LV scanning electron microscope.

### Snail sampling and shedding experiments

The study area constituted of Mwenje reservoir, and the northeastern shoreline of Lake Kariba, which are two artificial reservoirs of the Zambezi river basin in Zimbabwe. Mwenje dam was erected in 1970, covers roughly 5 km^2^ when full and has a catchment area of approximately 557 km^2^ [95]. The Kariba dam was completed in 1959 and Lake Kariba covers about 5,580 km^2^ when full and has an approximated catchment area of 815,000 km^2^ [96]. Sampling sites are displayed in Fig. 4. Locations sampled at Lake Kariba were adopted from prior studies [5, 79]. The Mwenje sampling site was located at S 17°14′ 47.9″ E 31°01′ 07.7″ in close vicinity to the reservoir. Snails were collected in October 2018 from water, aquatic vegetation, and sediment by two persons for 30 min per site, using both a scooping net and manual collection. Collected snails were stored in jars with lake water from the site of origin and kept at a constant temperature during transport. Upon arrival, snails were sorted per morphotype, counted, and preliminary identified based on the morphological identification keys of Brown [97] and Mandahl-Barth [98]. Next, shedding experiments were conducted: snails were separated in 12-well cell culture plates and incubated in filtered and aged lake water in complete darkness overnight, followed by bright light exposure for 5 h from 7 am to 12 pm. This treatment induces the natural emergence (i.e., *shedding*) of larval stages, the so-called cercariae, of several trematode species [55, 99]. Well contents and surfaces were inspected with a stereomicroscope for the presence of cercariae and/or metacercariae, respectively, as cercariae from liver flukes are known to rapidly encyst and form metacercariae on biotic or abiotic surfaces [100]. This was done at the start of the shedding experiment, 10 pm, 7 am, and hourly between 7 am and 12 pm during the bright light exposure experiment to detect nocturnal and diurnal shedding, respectively. All snails were sacrificed by heat shock in ~ 70 °C water to prevent contraction and simplify later DNA extraction, followed by fixation in 80% ethanol, and then we added the released cercariae to the same tube in the event of shedding. Snails that released cercariae were individually stored together with the emerged cercariae, while non-shedding snails were pooled per species per site. One representative of each snail morphotype per site was, prior to DNA extraction, fixed on black clay and photographed from its ventral and dorsal side according to Brecko et al. [94] using the focus stacking system described above. The obtained pictures were processed in Microsoft PowerPoint® by removing the image background and combining the front and rear perspective of the shell in one picture while ensuring a constant scale size. The (meta)cercariae were placed in 80% ethanol on a glass slide with a glass cover. For taking and compiling photos, we used a digital camera QImaging MicroPublisher 5.0 RTV mounted on a Leitz Dialux 22 stereomicroscope and piloted with the Syncroscopy’s Image Reconstruction Software Auto-Montage Pro (version 5.03.0061). The resulting images of (meta)cercariae were used for measurements in the ImageJ software (version 1.8.0).

### DNA extraction

DNA of ethanol preserved adult trematodes was extracted using the DNeasy Blood and Tissue kit (Qiagen™) according to the manufacturer’s protocol. For each DNA extraction, we used a small piece of tissue isolated from the posterior side of the specimen with a sterile scissor. The DNeasy Blood and Tissue kit (Qiagen™) was not successful for the museum samples of adult trematodes. The specimens are currently stored in 70% ethanol yet were initially fixed in formaldehyde at the time of collection. Formaldehyde, a storage medium often used in the past by museums, is known to inhibit proteinase *K-*based DNA extractions [101]. Therefore, Chelex® (Biorad™) and phenol chloroform isoamyl alcohol (PCI) based DNA extraction methods were assessed for the same museum samples, which do not rely on proteinase *K* for lysis. For the Chelex-based extraction, tissue was first incubated for 24 h in ATL buffer (Qiagen™) to flush excess formaldehyde as suggested by Shedlock et al. [101]. Next, samples were homogenized with a sterile scalpel on a glass plate. Tissue homogenate was incubated for 1 h at 56 °C and 30 min at 95 °C in 200 μL of 5% Chelex® (Biorad™) solution in a shaking Thermocycler™. Next, samples were centrifuged for 7 min at 13,000×*g*, and supernatant DNA extract was collected and stored at − 20 °C. Regarding the PCI-based DNA extraction, tissue samples were dried with sterile absorbent paper, 300 μL Tris-EDTA (TE) buffer and 300 μL PCI solution (phenol pH 6.7-chloroform-isoamylalcohol [25:24:1]) was added, and tissue was lysed by micro-bead shearing in a FastPrep-24^TM^ Classic lysis system (20 s, 6 m/s) (MP biomedical™). The DNA containing fraction was separated from lipids and cellular debris by 10 min of centrifugation at 14,000 rpm. Next, 15 μL sodium acetate (3 M, pH 5.2) and 450 μL ethanol (100%) were added and the mixture was placed in a freezer at − 20 °C for 20 min followed by centrifugation for 10 min at 4 °C (14,000 rpm) for DNA precipitation. Pellets were washed by adding ice cold ethanol (70%) followed by centrifugation (10 min at 4 °C, 14,000 rpm). The resulting DNA was dissolved in TE buffer and stored at − 20 °C. Successful DNA isolation was verified by gel electrophoresis (0.8% agarose gel, SYBR™ Safe).

The DNA of (meta)cercariae was extracted using a proteinase *K-*based lysis buffer as described in Ziętara et al. [102]. In short, 10 μL of the prepared proteinase *K* lysis buffer was added to a single cercaria in 10 μL of MilliQ water (Merck, Darmstadt, Germany). A first and second incubation step followed at 65 °C for 25 min and 95 °C at 10 min, respectively. After the protocol, DNA extracts were stored undiluted at − 20 °C.

For DNA extractions from ethanol preserved snail samples, soft tissue was isolated from the shell by using a sterile needle to make a hole in the apex to push the soft tissue out of the shell. Next, the isolated tissue was dried using absorbent paper and homogenized using a sterile scalpel. DNA was extracted using the E.Z.N.A.® Mollusc DNA Kit (OMEGA Bio-tek, Norcross, GA, USA) according to the manufacturer’s protocol and eluted through two elution steps of 75 μL, totaling to 150 μL of DNA extract. Additionally, the more cost- and time efficient Chelex® (Biorad™) method [5, 103] was used on 24 non-shedding *P. columella* specimens per site in Lake Kariba, as described previously. All DNA extracts were diluted 1:10 with MilliQ water (Merck, Darmstadt, Germany) to prevent inhibition of PCR reactions.

### Molecular diagnosis of infected snails

The DNA extracts of a maximum of 24 snails per species per site were subjected to the diagnostic multiplex PCR methods described in Schols et al. [42] and Carolus et al. [5]. These approaches enable rapid and cost-efficient diagnosis of trematode infections in snail DNA extracts, with simultaneous detection of *Schistosoma* spp. [42] or *Fasciola* spp. [5]. Both methods are based on the amplification of multiple amplicons of different lengths in a single PCR reaction: an internal control that amplifies *18S* snail rDNA to confirm the success of the DNA extraction and PCR reaction, a general trematode primer pair designed to amplify the *18S* rDNA of all trematode genera that have a reference sequence available in GenBank and S*chistosoma*- [42] or *Fasciola*- [5] specific internal transcribed spacer 2 (*ITS2*) or mitochondrial cytochrome c oxidase subunit I (*COI*) primers, respectively. All used primers and PCR protocols are described elsewhere [5, 42]. Samples that were positive for a *Schistosoma* spp. infection were subsequently analyzed using a second multiplex PCR according to the two-step approach described by Schols et al. [42]. This PCR differentiates between several schistosomes of veterinary and medical importance (i.e., *S. haematobium*, *S. mansoni*, *S. mattheei* and *S. bovis/S. curassoni/S. guineensis*) by generating species-specific *COI* amplicons of different lengths. By selectively screening all snail DNA extracts using either of the two methods (i.e., Lymnaeidae with the method of Carolus et al. [5] and Planorbidae with the method of Schols et al. [42]), we identified *Schistosoma* spp. and *Fasciola* spp. infections that were used for further analysis.

### PCR and DNA sequencing

A fragment of the mitochondrial *COI* and a nuclear rDNA region covering the *18S* rDNA marker, the internal transcribed spacer 1 (*ITS1*), the *5.8S* rDNA marker, the internal transcribed spacer 2 (*ITS2*), and/or the partial *28S* rDNA marker were amplified to assess the taxonomic position of trematode species sampled in this study. Additional file 1: Figure S4 indicates the annealing sites of the used primers for amplification of the rDNA region of adult liver flukes. The primers and resulting amplicon lengths are listed in Table 3, together with those for snail and trematode identification. We barcoded (*COI* sequencing) two random specimens per snail species and all *Schistosoma* and *Fasciola* spp. infected snails. For the parasites, we selected all *Schistosoma* and *Fasciola* spp. infections, all adult fasciolids and one adult amphistome specimen per morphotype. A new *Fasciola-*specific reverse primer COI_FAS_R (‘5-GACAAACAAACACAAGCAGG-3’), targeting a shorter *COI* fragment of 148 nucleotides when combined with the COI1_DIG_F primer (see Table 3), was designed to amplify DNA of the *Fasciola* museum samples, as no successful PCR amplification was obtained using the longer amplicon. It has become apparent that shorter amplicons (< 400 bp) outperform longer amplicons due to severe DNA fragmentation and/or degradation in old samples [107–109], such as the museum specimens in this study. Simplex PCR reactions and programs for sequencing were performed, according to Carolus et al. [5], in a 15 μL volume with 1.5 μL of DNA extract using the Qiagen™ Taq DNA polymerase kit containing 1.5 mM PCR buffer (Qiagen™), 0.6 mM dNTP mix (Qiagen™), 1.5 mM MgCl2, 0.45 units of Taq Polymerase (Qiagen™), 0.8 μM forward primer, and 0.8 μM reverse primer. The PCR reactions were performed as follows: initial denaturation at 94 °C for 15 min, 39 cycles of 94 °C for 30 s, annealing temperature depending on primer pair as listed in Table 3 for 45 s and 72 °C for 45 s and a final elongation step at 72 °C for 10 min in a Tprofessional Thermocycler (Biometra™). PCR products were visualized by gel electrophoresis on a 2% agarose gel with Midori Green Direct® staining and UV light. Samples that showed a clear amplified PCR product of the expected length were purified using the ExoSAP (Fermentas™) PCR purification protocol and sequenced using BigDye® chemistry (Macrogen™). The resulting sequences were processed (quality control, annotation, and generation of consensus sequences from forward and reverse primer sequencing) with Geneious® (R10). The *Trim Ends* command with an error probability limit of 5% in Geneious® was used to trim the obtained sequences. The complete chromatogram was inspected before and after consensus generation to resolve any additional ambiguities and ensure high sequence quality.

**Table 3:** Primers used to obtain mitochondrial (*COI*) and nuclear (*18S*, *ITS1*, *5.8S*, *ITS2*, and *28S*) amplicons for sequencing. The primer name is listed with the target organism, the targeted marker, the annealing temperature, the amplicon length, the primer sequence, and the literature reference from which primers were obtained. *COI* mitochondrial cytochrome c oxidase subunit 1, *ITS1* and *ITS2* internal transcribed spacer 1 and 2, respectively bp base pairs, and *F. nyanzae Fasciola nyanzae*. The rDNA region and *COI* marker of the metacercariae were amplified with the 18S_Dig_F–1270R, ITS4–ITS5, and COI1_Dig_F–NasmitR primer combinations

Primer name	Used on	Marker	Annealing Temp.	Length (bp)	Primer sequence (5′-3′)	Reference
WormA	*F. nyanzae*	*18S*	54 °C	1870	GCGAATGGCTCATTAAATCAG	[104]
WormB					CTTGTTACGACTTTTACTTCC	[104]
18S_Dig_F	*F. nyanzae*	*18S*	50 °C	1161	CAGCTATGGTTCCTTAGATCRTA	[5]
1270R					CCGTCAATTCCTTTAAGT	[105]
ITS5	*F. nyanzae*	*ITS1*-*5.8S*-*ITS2*	50 °C	1065	GGAAGTAAAAGTCGTAACAAG	[106]
ITS4					TCCTCCGCTTATTGATATGC	[106]
ITS2_Dig_F	*F. nyanzae*	*ITS2*-*28S*	50 °C	647	CAAHAAGTCGTGGMTTGG	Hammoud C, Mulero S, Van Bocxlaer B, Boissier J, Verschuren D, Albrecht C, Huyse T: Simultaneous genotyping of snails and infecting trematode parasites using high-throughput amplicon sequencing, forthcoming
ITS2_Dig_R					AACAACCCGACTCCAAGG	Hammoud C, Mulero S, Van Bocxlaer B, Boissier J, Verschuren D, Albrecht C, Huyse T: Simultaneous genotyping of snails and infecting trematode parasites using high-throughput amplicon sequencing, forthcoming
COI1_Dig_F	*F. nyanzae* and Stomach flukes	*COI*	53 °C	943	CNATGATNTTNTTTTTTTTRATGCC	Hammoud C, Mulero S, Van Bocxlaer B, Boissier J, Verschuren D, Albrecht C, Huyse T: Simultaneous genotyping of snails and infecting trematode parasites using high-throughput amplicon sequencing, forthcoming
Nasmit R					ACATAATGAAARTCAGCNAYMACRA	Hammoud C, Mulero S, Van Bocxlaer B, Boissier J, Verschuren D, Albrecht C, Huyse T: Simultaneous genotyping of snails and infecting trematode parasites using high-throughput amplicon sequencing, forthcoming
COI1_Dig_F	Museum fasciolids	*COI*	53 °C	148	CNATGATNTTNTTTTTTTTRATGCC	Hammoud C, Mulero S, Van Bocxlaer B, Boissier J, Verschuren D, Albrecht C, Huyse T: Simultaneous genotyping of snails and infecting trematode parasites using high-throughput amplicon sequencing, forthcoming
COI_FAS_R					GACAAACAAACACAAGCAGG	This study
Fasc-ITS1 F	*Fasciola* sp. from infected snails	*ITS1*-*5.8S*-*ITS2*	55 °C	716	TCTACTCTTACACAAGCGATACAC	[7]
Fasc-ITS1 R					GGCTTTCTGCCAAGACAAG	[7]
COI1_Dig_F	*Fasciola* and *Schistosoma* sp. from infected snails	*COI*	50 °C	451	CNATGATNTTNTTTTTTTTRATGCC	Hammoud C, Mulero S, Van Bocxlaer B, Boissier J, Verschuren D, Albrecht C, Huyse T: Simultaneous genotyping of snails and infecting trematode parasites using high-throughput amplicon sequencing, forthcoming
COI1_Dig_R					GMASWACCAAAWTTHCGATCAAA	Hammoud C, Mulero S, Van Bocxlaer B, Boissier J, Verschuren D, Albrecht C, Huyse T: Simultaneous genotyping of snails and infecting trematode parasites using high-throughput amplicon sequencing, forthcoming
ITS2_Schisto_F	*Schistosoma* sp. from infected snails	*ITS1-5.8S*-*ITS2*	62 °C	369	GGAAACCAATGTATGGGATTATTG	[42]
ITS2_Schisto_R					ATTAAGCCACGACTCGAGCA	[42]
COI1_snail_F	Snails	*COI*	50 °C	536	TAATTWATTGTTACDGCWCATGC	Hammoud C, Mulero S, Van Bocxlaer B, Boissier J, Verschuren D, Albrecht C, Huyse T: Simultaneous genotyping of snails and infecting trematode parasites using high-throughput amplicon sequencing, forthcoming
COI1_snail_R					CWCCTCCTGCWGGATCAAA	Hammoud C, Mulero S, Van Bocxlaer B, Boissier J, Verschuren D, Albrecht C, Huyse T: Simultaneous genotyping of snails and infecting trematode parasites using high-throughput amplicon sequencing, forthcoming

### Phylogenetic analysis

All sequences of sufficient quality were compared to the GenBank (https://blast.ncbi.nlm.nih.gov/) and BOLD (http://v3.boldsystems.org/) databases using the Basic Local Alignment Search Tool (BLAST) and BOLD Identification System (IDS), respectively. In case the species could not be clearly identified (i.e., no highly similar reference sequence or high identity matches to several species), a phylogenetic analysis was performed in which sequences of all closely related taxa, with an available sequence in the GenBank database, were aligned with the MUSCLE alignment algorithm [110] in AliView (version 1.26). Reference sequences of *Fasciola nyanzae*, *Tenuifasciola tragelaphi*, several amphistome families, and several members of the *Bulinus truncatus/tropicus* species complex [36] were absent from the database and could not be included in the analyses. The alignment was visually inspected for anomalies and trimmed to the largest possible consensus, and the model selection analysis was run in MEGA (version 10.2.5) to assess the suitability (i.e., best model or only marginally different AIC and BIC values from the best model) of the General Time Reversible (GTR) substitution model. This model proved suitable for all datasets with the exception of Lymnaeidae phylogeny, which required the Hasegawa-Kishino-Yano (KHY) substitution model. The DAMBE software (version 7.2.152, [111]) was used to assess substitution saturation in all datasets through the Xia’s [112] and Steel’s method [113] according to Xia and Lemey [114]. For the Steel’s method, mean phi values below 0.04 were assumed to lack phylogenetic information and were removed following Xia and Lemey [114]. For the *COI* phylogeny, we tested the datasets for saturation in (1) the first and second codon position and (2) the third codon position. For the fasciolid rDNA dataset, we only assessed substitution saturation for the non-coding *ITS1* and *ITS2* markers as *18S*, *5.8S*, and *28S* rDNA are slowly evolving markers. Possible saturation signals were evaluated by constructing a Maximum likelihood tree from DNAML in DAMBE and assessing the tree’s topology. The MEGA software was also used to estimate the corrected pairwise distances among *COI* sequences to correct for substitution saturation. For this, the Tamura-Nei (TN93) substitution model with gamma correction was applied. Gamma values were obtained through the model selection analysis in MEGA as mentioned above. A Bayesian inference of phylogeny was calculated using MrBayes (version 3.2.6) on the CIPRES portal (version 3.3) [115] (https://www.phylo.org/portal2) using the same model, for 10,000,000 generations while sampling the Markov chain every 1000 steps. The first 25% of the sampled values were discarded as burn-in and the analysis stopped if the convergence diagnostic fell below 0.01. Convergence was confirmed by checking whether the effective sampling size (ESS) was above 100 and by visually inspecting the absence of a trend in the convergence plots in MrBayes through the *sump* command and Tracer software (version 1.7.1). Finally, a maximum likelihood (ML) tree was also calculated through the IQ-tree web servers (http://iqtree.cibiv.univie.ac.at), and nodal support was assessed through 1000 bootstrap replicates [116]. The resulting trees were rooted on the respective outgroup in Figtree (version 1.4.4). The same software was used to create a consensus tree, containing nodal support values of Bayesian inference and ML phylogenies, that was exported in PDF format to Adobe Acrobat Pro DC (version 2020.012.20048), in which we adjusted the position of ill placed nodal support values and italicized species names. All markers, alignment lengths, and obtained sequences used for phylogenetic analyses are provided in their respective section in the results.

### Haplotype network of *Fasciola nyanzae*

A *COI* haplotype network based on 412 bp was constructed to study the genetic intraspecific variation of *Fasciola nyanzae*. The obtained *COI* sequences from two adult liver flukes and six snail infections were aligned with those obtained by Carolus et al. [5] (“GenBank: MK330623, MK330624, MK330625”) using the MUSCLE alignment algorithm [110] in AliView (version 1.26) and trimmed to the longest common overlapping sequence. Unique haplotypes were defined using the DnaSP® software (version 6.12) [117] and subsequently mapped in PopART® (version 1.7) (http://popart.otago.ac.nz) using the TCS network inference model [118].

## Supplementary Information

**Additional file 1: Figure S1**. SEM imaging of ‘Hippo stomach fluke type 1’. **Figure S2**. The morphometric approach to identify the hippo liver flukes. **Figure S3**. *F. nyanzae* metacercariae isolated from *P. columella*. **Figure S4**. Primers used to amplify the partial rDNA region of *F. nyanzae*. **Figure S5**. *COI*-based Phylogeny of the *Radix* genus. **Figure S6**. *COI*-based Phylogenies of the genera *Bulinus* and *Biomphalaria*. **Table S1**. Corrected *COI* genetic distances for the subfamily Fasciolinae. **Table S2**. Corrected rDNA genetic distances for the subfamily Fasciolinae. **Table S3**. Corrected *COI* genetic distances for the genus *Radix*. **Table S4**. Corrected *COI* genetic distances for the genus *Biomphalaria*. **Table S5**. Corrected *COI* genetic distances for the genus *Bulinus*. **Table S6**. Accession numbers generated in this study, Carolus et al. [5] and Muzarabani et al. (preprint, [33]).

## Data Availability

Lead contact Further information requests should be directed to and will be fulfilled by the lead contact, Ruben Schols (ruben.schols@africamuseum.be; ruben.schols@kuleuven.be). Materials availability DNA and tissue samples of all studied parasites, snails, and the culled *H. amphibius* are stored in the RMCA’s collection and are available with a completed Materials Transfer Agreement, with the exception of Hippo stomach fluke type 2 as the complete worms were used for DNA extraction. Data and code availability The sequences generated in this study have been deposited in the public repository NCBI GenBank under: MT909560-MT909561, MT909545-MT909550, MT909542-MT909543, MT893586-MT893595, MT888842-MT888847, MT886702-MT886703, and MT884914-MT884924. All GenBank identifiers generated by this study, and those of Carolus et al. [5] and Muzarabani et al. (preprint, [33]) involved in the analyses, are listed together with the sample type, marker, taxonomic identification, and region of origin in Additional file 1: Table S6. The sequence datasets used in this study are available in the supplementary file “Raw_data.”
