# Supplementary material for: Invasive snails, parasite spillback, and potential parasite spillover drive parasitic diseases of Hippopotamus amphibius in artificial lakes of Zimbabwe
Source: BMC Biol. 2021 Aug 20;19:160. doi: 10.1186/s12915-021-01093-2 (PMC8377832; doi:10.1186/s12915-021-01093-2)
Supplement: Supplementary file 1 — Additional file 1: Figure S1. SEM imaging of ‘Hippo stomach fluke type 1’. Figure S2. The morphometric approach to identify the hippo liver flukes. Figure S3. F. nyanzae metacercariae isolated from P. columella. Figure S4. Primers used to amplify the partial rDNA region of F. nyanzae. Figure S5. COI-based Phylogeny of the Radix genus. Figure S6. COI-based Phylogenies of the genera Bulinus and Biomphalaria. Table S1. Corrected COI genetic distances for the subfamily Fasciolinae. Table S2. Corrected rDNA genetic distances for the subfamily Fasciolinae. Table S3. Corrected COI genetic distances for the genus Radix. Table S4. Corrected COI genetic distances for the genus Biomphalaria. Table S5. Corrected COI genetic distances for the genus Bulinus. Table S6. Accession numbers generated in this study, Carolus et al. [5] and Muzarabani et al. (preprint, [33]). [file 12915_2021_1093_MOESM1_ESM.docx]

**Invasive snails, parasite spillback, and potential parasite spillover drive parasitic diseases of *Hippopotamus amphibius* in artificial lakes of Zimbabwe**

Ruben Schols^a,b,*,+^, Hans Carolus^c,*^, Cyril Hammoud^a,d^, Kudzai C. Muzarabani^e^, Maxwell Barson^e,f,g^ and Tine Huyse^a^

^a^ Department of Biology, Royal Museum for Central Africa, Tervuren, Belgium

^b^ Laboratory of Aquatic Biology, KU Leuven Kulak, Kortrijk, Belgium

^c^ Laboratory of Molecular Cell Biology, KU Leuven-VIB center for microbiology, Leuven, Belgium

^d^ Limnology research unit, Ghent University, Ghent, Belgium

^e^ Department of Biological Sciences, University of Zimbabwe, Harare, Zimbabwe

^f^ Department of Biological Sciences, University of Botswana, Gaborone, Botswana

^g^ Lake Kariba Research Station, University of Zimbabwe, Kariba, Zimbabwe

^*^ These authors contributed equally.

^+^ Lead Contact

Corresponding author: Ruben Schols (ruben.schols@africamuseum.be)

**Legend**

Additional file 1: Figures S1-S5. Figure S1 - SEM imaging of “Hippo stomach fluke type 1”. Figure S2 - The morphometric approach to identify the hippo liver flukes. Figure S3 - *F. nyanzae* metacercariae isolated from *P. columella*. Figure S4 – Primers used to amplify the partial rDNA region of *F. nyanzae*. Figure S5 – *COI*-based Phylogeny of the *Radix* genus. Figure S6 – *COI*-based Phylogenies of the genera *Bulinus* and *Biomphalaria*. Tables S1-S6. Table S1 – Corrected *COI* genetic distances for the subfamily Fasciolinae. Table S2 – Corrected rDNA genetic distances for the subfamily Fasciolinae. Table S3 – Corrected *COI* genetic distances for the genus *Radix*. Table S4 – Corrected *COI* genetic distances for the genus *Biomphalaria*. Table S5 – Corrected *COI* genetic distances for the genus *Bulinus*. Table S6 – Accession numbers generated in this study, Carolus et al. [5] and Muzarabani et al. (preprint, [33]).

**Supplemental Figures**





**Figure S1**: SEM imaging of the oral opening of “Hippo stomach fluke type 1”. The tegumental papillae are arranged in several rows and resemble the “*Dome to conical, nonciliated papilla*” described in Sey [57].


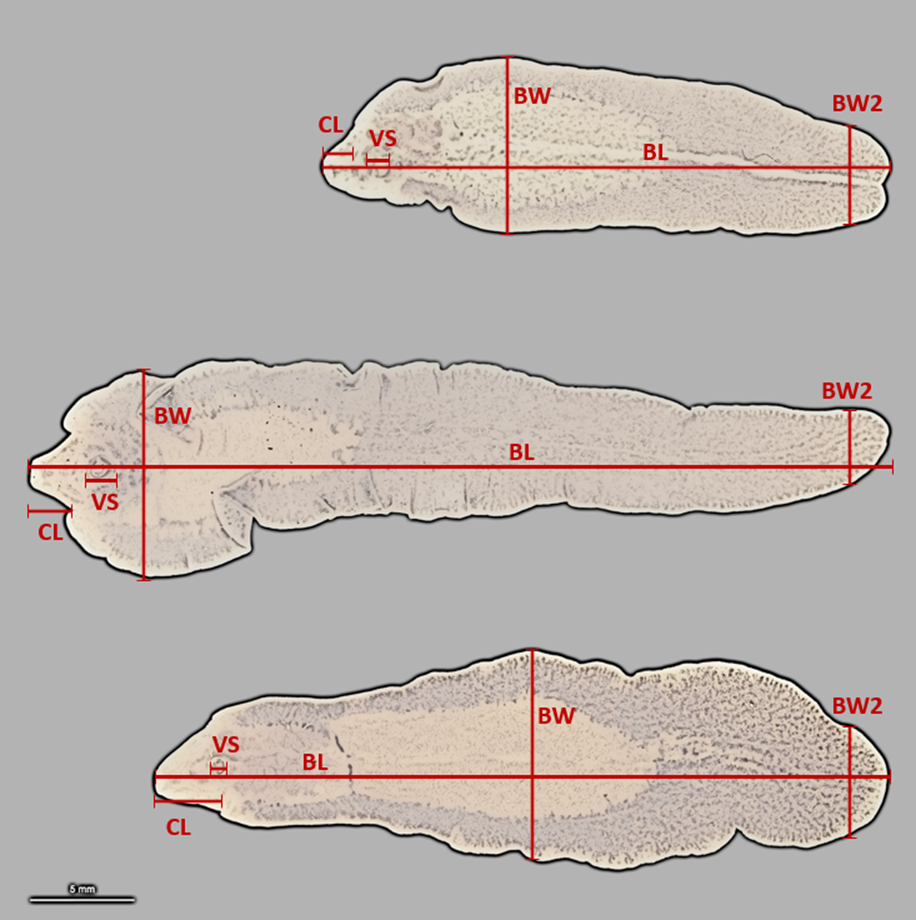


**Figure S2**: A visualization of the conducted measurements in the morphometric analysis of the hippo liver flukes. Samples included are, from top to bottom, *Fasciola hepatica*, *Fasciola nyanzae* and *Fasciola gigantica*. The following measurements were obtained: body width at widest point (BW), body length (BL), cephalic cone length until shoulders (CL), posterior width at 2 mm from the posterior end (BW2) and the anteroposterior diameter of the ventral sucker (VS). Scale bar represents 5 mm. A filter was added to the entire figure to enhance contrasts and improve interpretability of the conducted measurements.


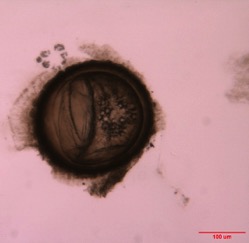

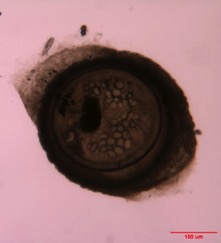


**Figure S3**: Encysted cercariae (i.e., metacercariae) used for morphometrics that encysted following emergence from two *Pseudosuccinea columella* snails from site 3 in Kariba. They are respectively listed under Hippo liver fluke 1 & 2 in **Table 1**. The scale bar represents 100 µm and accounts for both pictures.


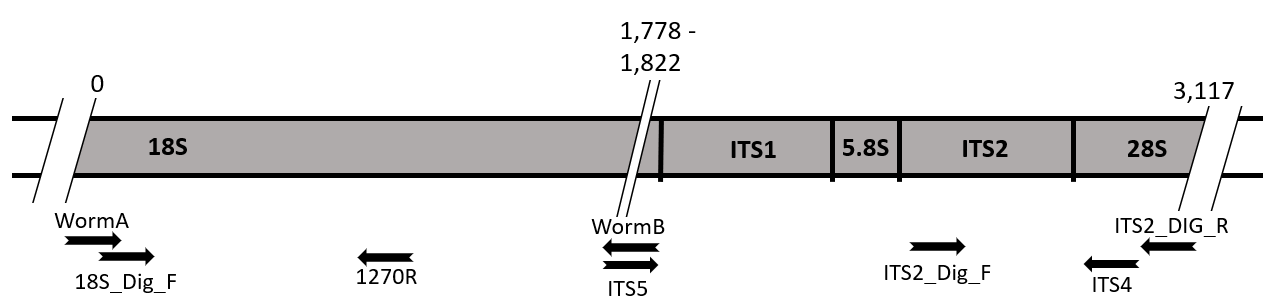


**Figure S4**: A visualization of the primers used for amplifying and sequencing the 3117 base pairs of the rDNA region of the Hippo liver flukes from Kariba. Base pair positions 1778 - 1822 are highlighted, as they could not be sequenced with the used primers. Primer information is listed in **Table 3**.





**Figure S5**: Maximum Likelihood analysis for the *Radix* species collected in this study, using the HKY + G (= 0.12) model on 463 bp of the *COI* marker. *Physella acuta* (“GenBank: NC_023253”) was used as an outgroup. Nodal support is indicated as bootstrap percentages (1000 bootstraps) and posterior probabilities, respectively before and after the “/” separator. GenBank accession numbers are provided after the “|” separator. Specimens from this study are indicated in blue.

**Figure S6**: A) Maximum Likelihood analysis for the *Biomphalaria* species collected in this study, using the GTR + G (= 0.19) model on 446 bp of the *COI* marker. *Planorbella duryi* (“GenBank: KY514384”), from the tribe Helisomatini, was used as an outgroup. B) Maximum Likelihood analysis for the *Bulinus* species collected in this study, using the GTR + G (= 0.19) model on 446 bp of the *COI* marker. *Indoplanorbis exustus* (“GenBank: KR811332”), from the subfamily Bulininae, was used as an outgroup. Nodal support is indicated as bootstrap percentages (1000 bootstraps) and posterior probabilities, respectively before and after the “/” separator. GenBank accession numbers are provided after the “|” separator. Specimens from this study are indicated in blue.


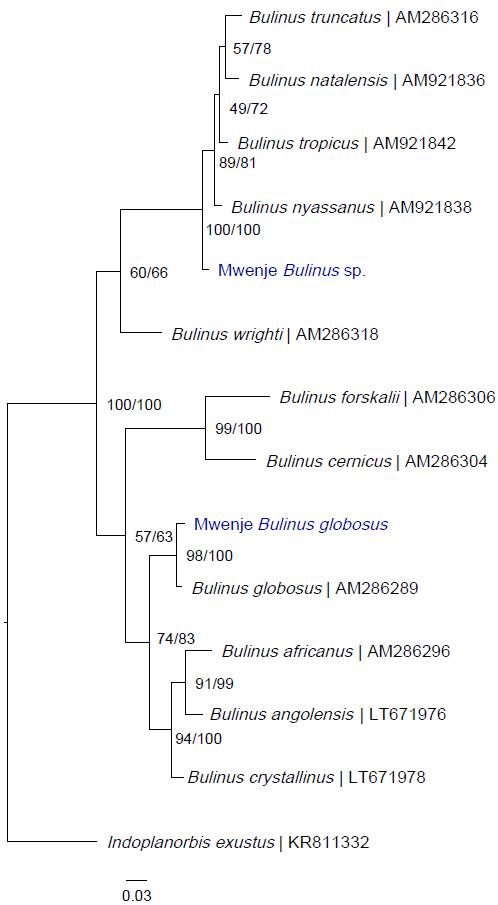

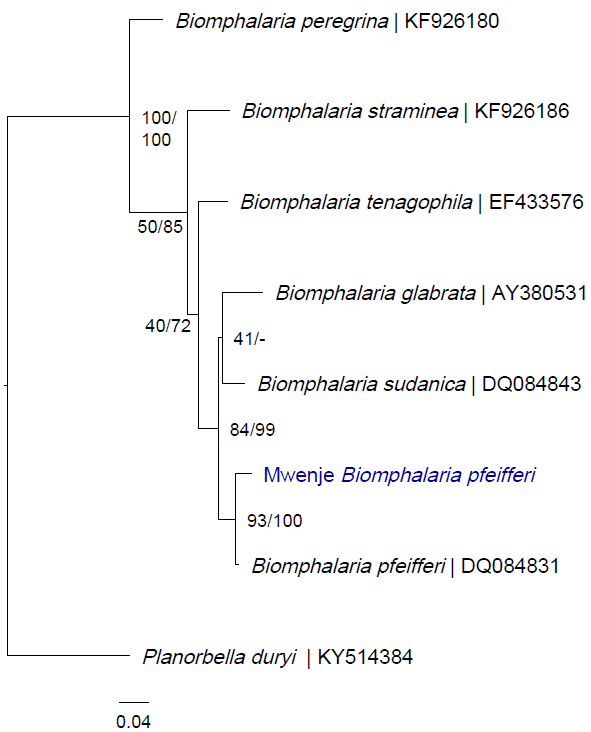


**A)**

**B)**

**Supplementary Tables**

**Table S1**: **Corrected *COI* genetic distances for the subfamily Fasciolinae.** Corrected genetic distances (Tamura-Nei model with gamma correction [G=0.23]) between members of the subfamily Fasciolinae based on a 814 bp *COI* fragment. All sequences obtained during this study are indicated in bold together with important p-distances relevant for species identification. GenBank accession numbers are provided after the “|” separator. *Fasciolopsis buski* (“GenBank: NC_030528”), from the subfamily Fasciolopsinae, was used as outgroup.

| Species |  | 1 | 2 | 3 | 4 | 5 | 6 |
| --- | --- | --- | --- | --- | --- | --- | --- |
| *Fasciolopsis buski* \| NC_030528 | 1 |  |  |  |  |  |  |
| *Fasciola jacksoni* \| KX787886 | 2 | 0,482 |  |  |  |  |  |
| *Fascioloides magna* \| EF534996 | 3 | 0,401 | 0,239 |  |  |  |  |
| *Fasciola hepatica* \| AP017707 | 4 | 0,291 | 0,331 | 0,224 |  |  |  |
| *Fasciola gigantica* \| NC_024025 | 5 | 0,358 | 0,248 | 0,244 | 0,168 |  |  |
| **Hippo liver fluke 1** | 6 | 0,401 | 0,328 | 0,225 | **0,180** | **0,144** |  |
| **Hippo liver fluke 2** | 7 | 0,343 | 0,284 | 0,206 | **0,164** | **0,106** | **0,025** |

**Table S2**: **Corrected rDNA genetic distances for the subfamily Fasciolinae.** Corrected genetic distances (Tamura-Nei model with gamma correction [G=0.05]) between members of the subfamily Fasciolinae based on 2771 bp of the rDNA region. All sequences obtained during this study are indicated in bold together with important p-distances relevant for species identification. GenBank accession numbers are provided after the “|” separator. *Fasciolopsis buski* (“GenBank: MN970005”), from the subfamily Fasciolopsinae, was used as outgroup.

| Species |  | 1 | 2 | 3 | 4 | 5 |
| --- | --- | --- | --- | --- | --- | --- |
| *Fasciolopsis buski* \| MN970005 | 1 |  |  |  |  |  |
| *Fasciola jacksoni* \| MN970006 | 2 | 0,113 |  |  |  |  |
| *Fascioloides magna* \| EF051080 | 3 | 0,155 | 0,034 |  |  |  |
| *Fasciola hepatica* \| MN970007 | 4 | 0,113 | 0,030 | 0,044 |  |  |
| *Fasciola gigantica* \| MN970009 | 5 | 0,109 | 0,030 | 0,046 | 0,005 |  |
| **Hippo liver fluke 1 & 2** | 6 | 0,107 | 0,031 | 0,045 | **0,002** | **0,004** |

**Table S3**: **Corrected *COI* genetic distances for the genus *Radix*.** Corrected genetic distances (Tamura-Nei model with gamma correction [G=0.16]) between *Radix* species based on a 463 bp *COI* fragment. All sequences obtained during this study are indicated in bold together with important p-distances relevant for species identification. GenBank accession numbers are provided after the “|” separator. *Physella acuta* (“GenBank: NC_023253”), from the family Physidae, was used as outgroup.

| Species |  | 1 | 2 | 3 | 4 | 5 | 6 | 7 | 8 | 9 | 10 | 11 | 12 | 13 | 14 | 15 | 16 | 17 | 18 | 19 | 20 | 21 |
| --- | --- | --- | --- | --- | --- | --- | --- | --- | --- | --- | --- | --- | --- | --- | --- | --- | --- | --- | --- | --- | --- | --- |
| *Physella acuta \|* NC_023253 | 1 |  |  |  |  |  |  |  |  |  |  |  |  |  |  |  |  |  |  |  |  |  |
| **Mwenje *Radix natalensis*** | 2 | 0,720 |  |  |  |  |  |  |  |  |  |  |  |  |  |  |  |  |  |  |  |  |
| **Kariba *Radix sp.*** | 3 | 0,702 | 0,363 |  |  |  |  |  |  |  |  |  |  |  |  |  |  |  |  |  |  |  |
| *Radix natalensis* \| HG977206 | 4 | 0,770 | **0,017** | 0,394 |  |  |  |  |  |  |  |  |  |  |  |  |  |  |  |  |  |  |
| *Radix rubiginosa* \| KY574609 | 5 | 0,683 | 0,361 | 0,396 | 0,325 |  |  |  |  |  |  |  |  |  |  |  |  |  |  |  |  |  |
| *Radix rubiginosa* \| KM067685 | 6 | 0,632 | 0,265 | 0,299 | 0,243 | 0,057 |  |  |  |  |  |  |  |  |  |  |  |  |  |  |  |  |
| *Radix rubiginosa* \| MH189925 | 7 | 0,678 | 0,269 | 0,309 | 0,243 | 0,058 | 0,020 |  |  |  |  |  |  |  |  |  |  |  |  |  |  |  |
| *Radix plicatula* \| MN737033 | 8 | 0,681 | 0,410 | **0,036** | 0,405 | 0,306 | 0,276 | 0,269 |  |  |  |  |  |  |  |  |  |  |  |  |  |  |
| *Radix plicatula* \| MN737034 | 9 | 0,704 | 0,426 | **0,040** | 0,421 | 0,318 | 0,287 | 0,277 | 0,002 |  |  |  |  |  |  |  |  |  |  |  |  |  |
| *Radix plicatula* \| MN737035 | 10 | 0,704 | 0,426 | **0,040** | 0,421 | 0,318 | 0,287 | 0,277 | 0,002 | 0,000 |  |  |  |  |  |  |  |  |  |  |  |  |
| *Radix dgebuadzei* \| MN718571 | 11 | 0,790 | 0,473 | 0,183 | 0,465 | 0,299 | 0,271 | 0,259 | 0,159 | 0,165 | 0,165 |  |  |  |  |  |  |  |  |  |  |  |
| *Radix dgebuadzei* \| MN718572 | 12 | 0,790 | 0,435 | 0,162 | 0,431 | 0,299 | 0,271 | 0,259 | 0,139 | 0,145 | 0,145 | 0,005 |  |  |  |  |  |  |  |  |  |  |
| *Radix euphratica* \| MN718574 | 13 | 0,660 | 0,472 | 0,125 | 0,437 | 0,337 | 0,265 | 0,265 | 0,091 | 0,095 | 0,095 | 0,101 | 0,091 |  |  |  |  |  |  |  |  |  |
| *Radix euphratica* \| MN718575 | 14 | 0,660 | 0,472 | 0,125 | 0,437 | 0,337 | 0,265 | 0,265 | 0,091 | 0,095 | 0,095 | 0,101 | 0,091 | 0,000 |  |  |  |  |  |  |  |  |
| *Radix euphratica* \| MN718576 | 15 | 0,660 | 0,472 | 0,125 | 0,437 | 0,337 | 0,265 | 0,265 | 0,091 | 0,095 | 0,095 | 0,101 | 0,091 | 0,000 | 0,000 |  |  |  |  |  |  |  |
| *Radix alticola* \| MH189954 | 16 | 0,603 | 0,397 | 0,210 | 0,392 | 0,333 | 0,345 | 0,333 | 0,171 | 0,178 | 0,178 | 0,119 | 0,119 | 0,099 | 0,099 | 0,099 |  |  |  |  |  |  |
| *Radix alticola* \| MH189964 | 17 | 0,541 | 0,384 | 0,157 | 0,381 | 0,293 | 0,277 | 0,266 | 0,124 | 0,129 | 0,129 | 0,088 | 0,076 | 0,076 | 0,076 | 0,076 | 0,020 |  |  |  |  |  |
| *Radix alticola* \| MN737036 | 18 | 0,586 | 0,373 | 0,181 | 0,370 | 0,277 | 0,253 | 0,237 | 0,125 | 0,130 | 0,130 | 0,107 | 0,093 | 0,094 | 0,094 | 0,094 | 0,032 | 0,014 |  |  |  |  |
| *Radix auricularia* \| KP242354 | 19 | 0,588 | 0,397 | 0,444 | 0,373 | 0,262 | 0,317 | 0,320 | 0,351 | 0,367 | 0,367 | 0,417 | 0,379 | 0,349 | 0,349 | 0,349 | 0,307 | 0,274 | 0,283 |  |  |  |
| *Radix auricularia* \| KP242340 | 20 | 0,588 | 0,397 | 0,444 | 0,373 | 0,262 | 0,317 | 0,320 | 0,351 | 0,367 | 0,367 | 0,417 | 0,379 | 0,349 | 0,349 | 0,349 | 0,307 | 0,274 | 0,283 | 0,000 |  |  |
| *Radix auricularia* \| KP242345 | 21 | 0,588 | 0,397 | 0,444 | 0,373 | 0,262 | 0,317 | 0,320 | 0,351 | 0,367 | 0,367 | 0,417 | 0,379 | 0,349 | 0,349 | 0,349 | 0,307 | 0,274 | 0,283 | 0,000 | 0,000 |  |
| *Radix makhrovi* \| MH189861 | 22 | 0,851 | 0,494 | 0,461 | 0,461 | 0,345 | 0,305 | 0,350 | 0,495 | 0,514 | 0,514 | 0,460 | 0,460 | 0,433 | 0,433 | 0,433 | 0,502 | 0,411 | 0,360 | 0,159 | 0,159 | 0,159 |

**Table S4: Corrected *COI* genetic distances for the genus *Biomphalaria*.** Corrected genetic distances (Tamura-Nei model with gamma correction [G=2.00]) between *Biomphalaria* species based on a 446 bp *COI* fragment. All sequences obtained during this study are indicated in bold together with important p-distances relevant for species identification. GenBank accession numbers are provided after the “|” separator. *Planorbella duryi* (“GenBank: KY514384”), from the tribe Helisomatini, was used as outgroup.

| Species |  | 1 | 2 | 3 | 4 | 5 | 6 | 7 |
| --- | --- | --- | --- | --- | --- | --- | --- | --- |
| **Mwenje *Biomphalaria* *pfeifferi*** | 1 |  |  |  |  |  |  |  |
| *Biomphalaria pfeifferi* \| DQ084831 | 2 | 0,**026** |  |  |  |  |  |  |
| *Biomphalaria peregrina* \| KF926180 | 3 | 0,142 | 0,136 |  |  |  |  |  |
| *Biomphalaria straminea* \| KF926186 | 4 | 0,120 | 0,105 | 0,135 |  |  |  |  |
| *Biomphalaria tenagophila* \| EF433576 | 5 | 0,089 | 0,073 | 0,137 | 0,095 |  |  |  |
| *Biomphalaria glabrata* \| AY380531 | 6 | 0,080 | 0,078 | 0,161 | 0,121 | 0,095 |  |  |
| *Biomphalaria sudanica* \| DQ084843 | 7 | 0,067 | 0,056 | 0,149 | 0,112 | 0,076 | 0,076 |  |
| *Planorbella duryi \|* KY514384 | 8 | 0,222 | 0,219 | 0,230 | 0,225 | 0,197 | 0,245 | 0,218 |

**Table S5: Corrected *COI* genetic distances for the genus *Bulinus*.** Corrected genetic distances (Tamura-Nei model with gamma correction [G=0.21]) between *Bulinus* species based on a 446 bp *COI* fragment. All sequences obtained during this study are indicated in bold together with important p-distances relevant for species identification. GenBank accession numbers are provided after the “|” separator. *Indoplanorbis exustus* (“GenBank: KR811332”), from the subfamily Bulininae, was used as outgroup.

| Species |  | 1 | 2 | 3 | 4 | 5 | 6 | 7 | 8 | 9 | 10 | 11 | 12 | 13 |
| --- | --- | --- | --- | --- | --- | --- | --- | --- | --- | --- | --- | --- | --- | --- |
| **Mwenje *Bulinus* *globosus*** | 1 |  |  |  |  |  |  |  |  |  |  |  |  |  |
| **Mwenje *Bulinus* sp.** | 2 | 0,252 |  |  |  |  |  |  |  |  |  |  |  |  |
| *Bulinus forskalii* \| AM286306 | 3 | 0,282 | 0,310 |  |  |  |  |  |  |  |  |  |  |  |
| *Bulinus cernicus* \| AM286304 | 4 | 0,227 | 0,333 | 0,179 |  |  |  |  |  |  |  |  |  |  |
| *Bulinus truncatus* \| AM286316 | 5 | 0,289 | **0,045** | 0,316 | 0,329 |  |  |  |  |  |  |  |  |  |
| *Bulinus natalensis* \| AM921836 | 6 | 0,274 | **0,031** | 0,359 | 0,304 | 0,038 |  |  |  |  |  |  |  |  |
| *Bulinus nyassanus*\| AM921838 | 7 | 0,266 | **0,029** | 0,334 | 0,319 | 0,044 | 0,036 |  |  |  |  |  |  |  |
| *Bulinus tropicus* \| AM921842 | 8 | 0,290 | **0,031** | 0,350 | 0,314 | 0,038 | 0,030 | 0,022 |  |  |  |  |  |  |
| *Bulinus wrighti* \| AM286318 | 9 | 0,164 | 0,173 | 0,296 | 0,242 | 0,199 | 0,197 | 0,180 | 0,191 |  |  |  |  |  |
| *Bulinus globosus* \| AM286289 | 10 | **0,015** | 0,245 | 0,280 | 0,244 | 0,298 | 0,283 | 0,276 | 0,300 | 0,153 |  |  |  |  |
| *Bulinus africanus* \| AM286296 | 11 | 0,134 | 0,288 | 0,308 | 0,254 | 0,281 | 0,306 | 0,289 | 0,289 | 0,213 | 0,133 |  |  |  |
| *Bulinus angolensis* \| LT671976 | 12 | 0,127 | 0,259 | 0,276 | 0,261 | 0,289 | 0,281 | 0,265 | 0,280 | 0,193 | 0,119 | 0,063 |  |  |
| *Bulinus crystallinus* \| LT671978 | 13 | 0,091 | 0,208 | 0,226 | 0,258 | 0,264 | 0,252 | 0,245 | 0,252 | 0,167 | 0,084 | 0,066 | 0,054 |  |
| *Indoplanorbis exustus* \| KR811332 | 14 | 0,316 | 0,369 | 0,432 | 0,407 | 0,474 | 0,395 | 0,435 | 0,409 | 0,330 | 0,284 | 0,332 | 0,340 | 0,289 |

**Table S6:** Accession numbers generated in this study, Carolus et al. [5] and Muzarabani et al. (preprint, [33]). Table listing all the GenBank accession numbers (Acc. Nr.) generated by this study and those included from Carolus et al. [5] and Muzarabani et al. (preprint, [33]). The table also lists information on the sample type and/or preliminary identification (Sample), the sequenced marker (Marker), the current identification (ID), the original locality (Locality) and an identifier to show which were generated in this study and which in Carolus et al. [5] or Muzarabani et al. (preprint, [33]). The ‘(partial)’ nominator is used to indicate that the sequence only spans part of that genetic marker.

| Acc. Nr. | Sample | Marker | ID | Locality | Reference |
| --- | --- | --- | --- | --- | --- |
| MT884914 | *Schistosoma* sp. infection from snail | *28S* (partial)*-ITS1*-*5.8S*-*ITS2-18S* (partial) | *S. haematobium* | Mwenje | This study |
| MT884915 | *Schistosoma* sp. infection from snail | *ITS1* (partial)-*5.8S*-*ITS2* (partial) | *S. edwardiense* | Mwenje | This study |
| MT884916 | *Schistosoma* sp. infection from snail | *ITS1* (partial)-*5.8S*-*ITS2* (partial) | *S. edwardiense* | Mwenje | This study |
| MT884917 | *Schistosoma* sp. infection from snail | *ITS1* (partial)-*5.8S*-*ITS2* (partial) | *S. edwardiense* | Mwenje | This study |
| MT884918 | *Schistosoma* sp. infection from snail | *ITS1* (partial)-*5.8S*-*ITS2* (partial) | *S. edwardiense* | Mwenje | This study |
| MT884919 | *Schistosoma* sp. infection from snail | *ITS1* (partial)-*5.8S*-*ITS2* (partial) | *S. edwardiense* | Mwenje | This study |
| MT884920 | *Schistosoma* sp. infection from snail | *ITS1* (partial)-*5.8S*-*ITS2* (partial) | *S. edwardiense* | Mwenje | This study |
| MT884921 | *Schistosoma* sp. infection from snail | *ITS1* (partial)-*5.8S*-*ITS2* (partial) | *S. edwardiense* | Mwenje | This study |
| MT884922 | *Schistosoma* sp. infection from snail | *ITS1* (partial)-*5.8S*-*ITS2* (partial) | *S. edwardiense* | Mwenje | This study |
| MT884923 | *Schistosoma* sp. infection from snail | *ITS1* (partial)-*5.8S*-*ITS2* (partial) | *S. edwardiense* | Mwenje | This study |
| MT884924 | *Schistosoma* sp. infection from snail | *ITS1* (partial)-*5.8S*-*ITS2* (partial) | *S. edwardiense* | Mwenje | This study |
| MT886702 | *Schistosoma* sp. infection from snail | *COI* (partial) | *S. edwardiense* | Mwenje | This study |
| MT886703 | *Schistosoma* sp. infection from snail | *COI* (partial) | *S. haematobium* | Mwenje | This study |
| MT888842 | snail | *COI* (partial) | *P. columella* | Kariba | This study |
| MT888843 | snail | *COI* (partial) | *Bulinus sp.* | Mwenje | This study |
| MT888844 | snail | *COI* (partial) | *B. globosus* | Mwenje | This study |
| MT888845 | snail | *COI* (partial) | *B. pfeifferi* | Mwenje | This study |
| MT888846 | snail | *COI* (partial) | *R. natalensis* | Mwenje | This study |
| MT888847 | snail | *COI* (partial) | *R.* aff. *plicatula* | Kariba | This study |
| MT893586 | *Fasciola* sp. infection from snail | *28S* (partial)*-ITS1*-*5.8S*-*ITS2-18S* (partial) | *F. nyanzae* | Mwenje | This study |
| MT893587 | *Fasciola* sp. infection from snail | *28S* (partial)*-ITS1*-*5.8S*-*ITS2-18S* (partial) | *F. nyanzae* | Mwenje | This study |
| MT893588 | *Fasciola* sp. infection from snail | *28S* (partial)*-ITS1*-*5.8S*-*ITS2-18S* (partial) | *F. nyanzae* | Mwenje | This study |
| MT893589 | *Fasciola* sp. infection from snail | *ITS1*-*5.8S*-*ITS2* | *F. nyanzae* | Mwenje | This study |
| MT893590 | *Fasciola* sp. infection from snail | *ITS1*-*5.8S*-*ITS3* | *F. nyanzae* | Mwenje | This study |
| MT893591 | *Fasciola* sp. infection from snail | *ITS1*-*5.8S*-*ITS4* | *F. nyanzae* | Mwenje | This study |
| MT893592 | *Fasciola* sp. infection from snail | *ITS1*-*5.8S*-*ITS5* | *F. nyanzae* | Mwenje | This study |
| MT893593 | *Fasciola* sp. infection from snail | *ITS1*-*5.8S*-*ITS6* | *F. nyanzae* | Mwenje | This study |
| MT893594 | *Fasciola* sp. infection from snail | *ITS1*-*5.8S*-*ITS7* | *F. nyanzae* | Mwenje | This study |
| MT893595 | *Fasciola* sp. infection from snail | *28S* (partial)*-ITS1*-*5.8S*-*ITS2-18S* (partial) | *F. nyanzae* | Mwenje | This study |
| MT909542 | Hippo liver fluke 1 | *COI* (partial) | *F. nyanzae* | Kariba | This study |
| MT909543 | Hippo liver fluke 2 | *COI* (partial) | *F. nyanzae* | Kariba | This study |
| MT909545 | *Fasciola* sp. infection from snail | *COI* (partial) | *F. nyanzae* | Kariba | This study |
| MT909546 | *Fasciola* sp. infection from snail | *COI* (partial) | *F. nyanzae* | Mwenje | This study |
| MT909547 | *Fasciola* sp. infection from snail | *COI* (partial) | *F. nyanzae* | Mwenje | This study |
| MT909548 | *Fasciola* sp. infection from snail | *COI* (partial) | *F. nyanzae* | Mwenje | This study |
| MT909549 | *Fasciola* sp. infection from snail | *COI* (partial) | *F. nyanzae* | Kariba | This study |
| MT909550 | *Fasciola* sp. infection from snail | *COI* (partial) | *F. nyanzae* | Mwenje | This study |
| MT909560 | Hippo stomach fluke Type 1 | *COI* (partial) | *C. cruciformis* | Kariba | This study |
| MT909561 | Hippo stomach fluke Type 2 | *COI* (partial) | Unidentified amphistome species | Kariba | This study |
| MT909820 | Hippo liver fluke 1 | *28S* (partial)*-ITS1*-*5.8S*-*ITS2-18S* (partial) | *F. nyanzae* | Kariba | This study |
| MT909821 | Hippo liver fluke 2 | *28S* (partial)*-ITS1*-*5.8S*-*ITS2-18S* (partial) | *F. nyanzae* | Kariba | This study |
| MK330628 | 99,3% match to MT909543 | *COI* (partial) | *F. nyanzae* | Kariba | Carolus et al. [5] |
| MK330629 | 99,3% match to MT909543 | *COI* (partial) | *F. nyanzae* | Kariba | Carolus et al. [5] |
| MK330630 | 99,3% match to MT909542 | *COI* (partial) | *F. nyanzae* | Kariba | Carolus et al. [5] |
| MK330623 | 99.9% match to MT909820&MT909821 | *ITS1* (partial)-*5.8S*-*ITS2* (partial) | *F. nyanzae* | Kariba | Carolus et al. [5] |
| MK330624 | 99.9% match to MT909820&MT909821 | *ITS1* (partial)-*5.8S*-*ITS2* (partial) | *F. nyanzae* | Kariba | Carolus et al. [5] |
| MK330625 | 99.9% match to MT909820&MT909821 | *ITS1* (partial)-*5.8S*-*ITS2* (partial) | *F. nyanzae* | Kariba | Carolus et al. [5] |
| MK333465 | *P. columella* | *COI* (partial) | *P. columella* | Kariba | Carolus et al. [5] |
| MK333466 | *Radix* sp. | *COI* (partial) | *R.* aff. *plicatula* | Kariba | Carolus et al. [5] |
| MT013349 | *B. truncatus* | *COI* (partial) | *B. truncatus* | Kariba | Muzarabani et al. (preprint, [33]) |
| MT013355 | *Carmyerius* sp. | *COI* (partial) | *C. cruciformis* | Kariba | Muzarabani et al. (preprint, [33]) |
| MT013350 | Unidentified amphistome species | *COI* (partial) | Unidentified amphistome species | Kariba | Muzarabani et al. (preprint, [33]) |
